# Therapeutic potential of zinc oxide/berberine nanoparticles in mitigating acute respiratory distress syndrome: in vivo and in silico approaches

**DOI:** 10.1186/s40360-025-01036-5

**Published:** 2025-12-01

**Authors:** Maysara S. El-Salakawy, Alshimaa A. Abd-Elmoneam, Mohammed S. Nofal, Nourhan M. Kolaib, Amany A. Elkashef, Ahmed Z. Ghareeb, Doaa A. Ghareeb

**Affiliations:** 1 Bioscreening and Preclinical Trials Lab, Biochemistry Department, Faculty of Science, Alexandria University, Alexandria, Egypt; 2 Clinical Pathology Department, Faculty of Medicine, Alexandria University, Alexandria, Egypt; 3 Chemistry Department, Faculty of Science, Alexandra University, Alexandria, Egypt; 4 Center of Excellence for Drug Preclinical Studies (CE-DPS), Pharmaceutical and Fermentation Industry Development Center, City of Scientific Research & Technological Applications (SRTA-City), New Borg El Arab, Alexandria, Egypt; 5 Research Projects Unit, Pharos University in Alexandria, Canal El Mahmoudia Street, Beside Green Plaza Complex, Alexandria, 21648 Egypt

**Keywords:** TREM-1, Reactive oxygen species, Neutrophil infiltration, Lung inflammation, Angiotensin-converting enzyme-2, Myeloperoxidase

## Abstract

**Background:**

Inflammation and oxidative stress strongly contribute to the pathophysiology of acute respiratory distress syndrome (ARDS), which is a life-threatening pulmonary disease. Zinc oxide/berberine nanoparticles (ZnO/Ber-NPs) have been shown to have a protective effect against COVID-19 because of their antioxidant, anti-inflammatory, and antiviral properties. Hence, this study aimed to investigate the therapeutic action of ZnO/Ber NPs against ARDS. ARDS was induced by a combination of lipopolysaccharides and nicotine (LPS + Nt).

**Methods:**

Male mice were induced with LPS + Nt alternately for 14 days and then orally administered berberine (Ber) (1.24 mg/kg), ZnO NPs (2.06 mg/kg), a ZnO NP + Ber mixture (3.3 mg/kg), or ZnO/Ber NPs (3.3 mg/kg). Assessment of (1) prooxidant and antioxidant (enzymatic and nonenzymatic) parameters, (2) inflammatory and anti-inflammatory markers (TNF-α, IL-1β, IFN-ɣ, NF-kB and IL-10), (3) lung lesion parameters, triggering receptor expressed on myeloid cells-1 (TREM-1), myeloperoxidase enzyme (MPO) and angiotensin-converting enzyme-2 (ACE II), (4) and apoptotic markers (Bax and p53) were performed via standardized methods. In addition, ZnO, Ber, and ZnO/Ber NPs were examined for their bioactivities against the following proteins GPx, code: 2R37, SOD protein, code: 1PL4, ACE2 protein, code: 6M1D, TREM1 protein, code: 1Q8M and MPO protein, code: 6WYZ.

**Results:**

The results demonstrated that LPS + Nt administration significantly elevated oxidative stress, proinflammatory, lung lesion, and proapoptotic parameters while reducing antioxidant and ACE II levels in the lung. Treatments, especially ZnO/Ber NPs, significantly attenuated the oxidative stress and inflammation associated with ARDS, as indicated by the restoration of antioxidant enzyme activities, decreased lipid peroxidation, and proinflammatory TNF-α and IFN-ɣ levels. ZnO/Ber NPs significantly decreased TREM-1 and MPO in association with elevated ACE II. In addition, ZnO/Ber NPs decreased the expression of apoptotic markers and decreased the number of alveolar inflammatory infiltrates to the minimal score. Molecular docking analysis revealed that ZnO/Ber NPs showed the strongest binding with all tested receptors.

**Conclusion:**

ZnO/Ber NPs exhibit antioxidant, anti-apoptotic, and anti-inflammatory effects and act as therapeutic candidates against ARDS.

**Supplementary Information:**

The online version contains supplementary material available at 10.1186/s40360-025-01036-5.

## Background

Berberine (Ber), an isoquinoline alkaloid, is predominantly found in the roots, stems, and bark of various plants belonging to the *Berberidaceae* family [1, 2]. For over 400 years, berberine has been a staple in traditional medicine across China, India, and the Middle East due to its various biological activities, such as antibacterial, anticancer, antimicrobial, antidiabetic, and antioxidant activities [3]. NPs have garnered significant interest because of their precise cell-targeting capabilities, excellent biocompatibility, adjustable biological activity, and superior pharmacokinetic properties. NPs can potentially evolve into a new class of therapeutic agents for managing inflammation. One of the most widely used types of nanoparticles is zinc oxide nanoparticles (ZnO NPs) because of their biocompatibility, easy synthesis, and low cost [4].

Despite its potential pharmacological benefits, Ber has poor solubility and bioavailability. Similarly, ZnO NPs exhibit some toxicity, which reduces their use as individual therapeutic agents. Hence, researchers have developed berberine-based ZnO NPs that overcome these disadvantages and benefit significantly from their pharmacological properties. The ZnO/Ber complex has been demonstrated to possess anticancer and antioxidant activities. Our group conducted a study to examine the activity of the ZnO/Ber complex against COVID-19 and its toxicity in vitro and in vivo [1]. The study revealed that the nanocomplex has a promising safety profile where it showed normal CBC parameters, and lower triglycerides level than normal control mice and prevent the toxic effect occurred to RBCs and WBCs due to hydroxychloroquine administration. Moreover, it showed normal AST, ALT, urea, uric acid, and creatinine levels, suggesting a protective effect of ZnO/Ber NPs against liver and kidneys toxicity. COVID-19 pathophysiology involves virus entry through ACE II, which induces oxidative stress and the NF-κB pathway, ending with a cytokine storm. The results showed that the complex inhibited viral entry by binding ACE II and modulating its binding with the virus receptor binding domain (RBD). Moreover, it has the highest antioxidant capacity and possesses anti-inflammatory activity by reducing TNF-α and IL-6. Hence, the ZnO/Ber complex could be a strong candidate for attenuating acute respiratory distress syndrome (ARDS) and its associated conditions.

ARDS is a heterogeneous pulmonary disease that is characterized by acute-onset respiratory failure caused by pneumonia, sepsis, and, recently, COVID-19 infection [5–7]. The pathophysiology of ARDS includes hyperinflammation, cytokine storms, oxidative stress, edema, and hypoxemia. In addition to epithelial and endothelial damage, hyperactivation and recruitment of neutrophils into injured lungs and hyaline membrane formation occur [7, 8]. ARDS is considered a fatal complication of COVID-19. Most patients admitted to intensive care units suffer from COVID-19-induced ARDS, with a global mortality rate between 23% and 56% over the past four years [9].

Oxidative stress and inflammatory responses consistently reinforce one another throughout the development of acute lung injury (ALI) [10]. A widely recognized approach for investigating ARDS is the use of a mouse model that induces ALI/ARDS through lipopolysaccharide (LPS) administration [11, 12]. LPS is a key component of the cell walls of gram-negative bacteria, through which the immune system is involved in bacterial infection [12]. As a result of the toll-like receptor 4 (TLR4) host immune response, the levels of proinflammatory cytokines and malondialdehyde (MDA) are elevated, whereas the levels of antioxidant enzymes are reduced in the context of LPS-induced lung injury [12]. In addition, nicotine is a natural alkaloid that is abundant in several plants and serves as the primary constituent of both e-cigarettes and tobacco [13]. Cigarette smoke is a major risk factor for ARDS. Research has indicated that short-term or long-term exposure to CS increases the severity of ARDS caused by LPS. CS negatively affects the immune system; intensifies macrophage and neutrophil infiltration in the lungs; increases pulmonary and endothelial permeability; and increases the production of proinflammatory mediators such as IL-1β, IL-6, TNF-α, IL-8, and ROS, leading to the destruction of alveoli and lung tissue [14, 15]. Nicotine causes acute lung inflammation, damage to the lung endothelial barrier, and disruption of the oxidative status of lung tissues [16]. The activation of the nuclear factor-kappa B (NF-κB) signaling pathway by LPS and nicotine induces oxidative stress and inflammation, as demonstrated by Aslan et al. [17] and Zhang et al. [18].

Understanding the immunogenic lung microenvironment reveals the inflammatory status associated with LPS-induced ARDS. Research conducted by Liu et al. [19] and Masterson et al. [20] demonstrated that the interplay between lung macrophages and neutrophils plays a significant role in the progression of ARDS. In particular, macrophages in lung tissue have pattern recognition receptors that recognize damage-associated molecular patterns (DAMPs), such as high-mobility group box 1 (HMGB1), and DNA and pathology-associated molecular patterns (PAMPs), such as LPS. When stimulated, these cells become active and polarize into classically activated macrophages (M1). As a result, they release tumor necrosis factor alpha (TNF-α), interleukins (IL), and chemoattractants that attract neutrophils and leukocytes, initiating an innate immune response in inflamed lungs [21]. Neutrophils migrate to alveoli from the surrounding circulation through attachment to the endothelial tissue. Endothelial cells activate sequestered neutrophils and bind to intercellular adhesion molecules (ICAM-1) from the endothelium. Activated neutrophils produce neutrophil extracellular traps (NETs), proinflammatory cytokines, and reactive oxygen species (ROS) that increase inflammation. Excessive activation and infiltration of neutrophils into the lungs induce alveolar damage and lung dysfunction [19, 22].

This study aimed to examine the antioxidant and anti-inflammatory properties of ZnO/Ber NPs to determine their potential as a new treatment approach for treating ARDS induced in a mouse model by LPS plus nicotine.

## Materials and methods

### Reagents, kits, and antibodies

Zinc oxide nanoparticles (NanoResearch Laboratory, H-21 Gopalpur, Jamshedpur, Jharkhand, India), trichloro acetic acid (Smart Lab, 9–11 BSD Sektor XI Tangerang Selatan, Indonesia), and 5,5-dithio-bis-(2-nitrobenzoic acid) (Sigma‒Aldrich, St. Louis, MO, USA) were used in this study. Commercial nicotine was purchased from the 007 team, Egypt. ELISA kits for mouse IL-10 (In-Mo1242), mouse IL-1β (In-Mo1256), mouse IFN-γ (In-Mo1233), and mouse TNF-α (In-Mo1920) were purchased from INNOVA Biotech, Daxing District, Beijing, China. Anti-rabbit IgG was purchased from Boster Biological Technology, Pleasanton, CA 94,566, USA. Anti-myeloperoxidase (MPO) (#79623), anti-angiotensin converting enzyme II (ACE II) (#15983), and anti-triggering receptor expressed on myeloid cells 1 (TREM-1) antibodies were purchased from Cell Signaling Technology (Beverly, MA, USA). TRIzol reagent was purchased from Thermo Fisher Scientific, Inc., Waltham, MA, USA. All other chemicals and solvents were of analytical grade.

### Methods

#### Preparation and characterization of ZnO/Ber NPs

A 1:1 ZnO/berberine complex (ZnO/Ber) was prepared via a facile mixing method with ratios reported in Ghareeb et al. [1]. A 5 mg/mL solution of ZnO nanoparticles or Ber in Milli-Q water was prepared via sonication for 30 min–10 min, respectively, via a probe-type sonicator. Ber solution was added wisely to the ZnO suspension under continuous vigorous stirring at room temperature for 2 h. Afterward, the mixture was concentrated via a vacuum evaporator at 50 °C, followed by lyophilization to collect excellent yellowish ZnO/Ber-NPs powder. This powder was reconstituted in Milli-Q water for further use and characterization. The synthesized ZnO/Ber complex was analyzed via Fourier transform infrared spectroscopy (FTIR), transmission electron microscopy (TEM), and zetasizer as detailed in our earlier study [1].

#### LPS extraction

LPS extraction was performed according to the method reported by Abdel-Latif et al. [23]. LPS was extracted from *E. coli* bacteria grown in Luria‒Bertani (LB) broth via a simple nonphenolic method involving methanol‒chloroform extraction.

#### HPLC for commercial nicotine

HPLC analysis was performed on an Agilent 1260 system with a diode array detector. The compounds were separated on a C18 column via an isocratic mobile phase composed of 0.1% triethylamine in water buffer and acetonitrile (70:30 ratio) at pH 7.0 under ambient temperature conditions. A flow rate of 1 mL/min was applied, and a gradient elution program was employed to achieve separation. The system was set to detect compounds at a wavelength of 254 nm. The sample, including the nicotine standard, was injected in 5 µl volumes [24].

#### Animals

A total of 60 male albino mice, with weights ranging from 20 to 25 g, were acquired from the Theodor Bilharz Research Institute in Cairo, Egypt. The mice were housed in the animal facility of the Pharmaceutical and Fermentation Industries Development Centre (PFIDC), SRTA-City. The animals were randomly divided into 10 groups (six mice/group) maintained in individual cages at 25 ± 2 °C. The mice were also subjected to a consistent 12:12-hour cycle of daylight and darkness. The mice were provided unrestricted access to water and a nutritionally balanced commercial diet. The protocol for animal experiments was approved by the Institutional Animal Care and Use Committee (IACUC) of Alexandria University’s animal research ethics committee. (IACUC)/AU # 04-22-11-26-1-01.

#### Experimental design

**Fig. 1:**
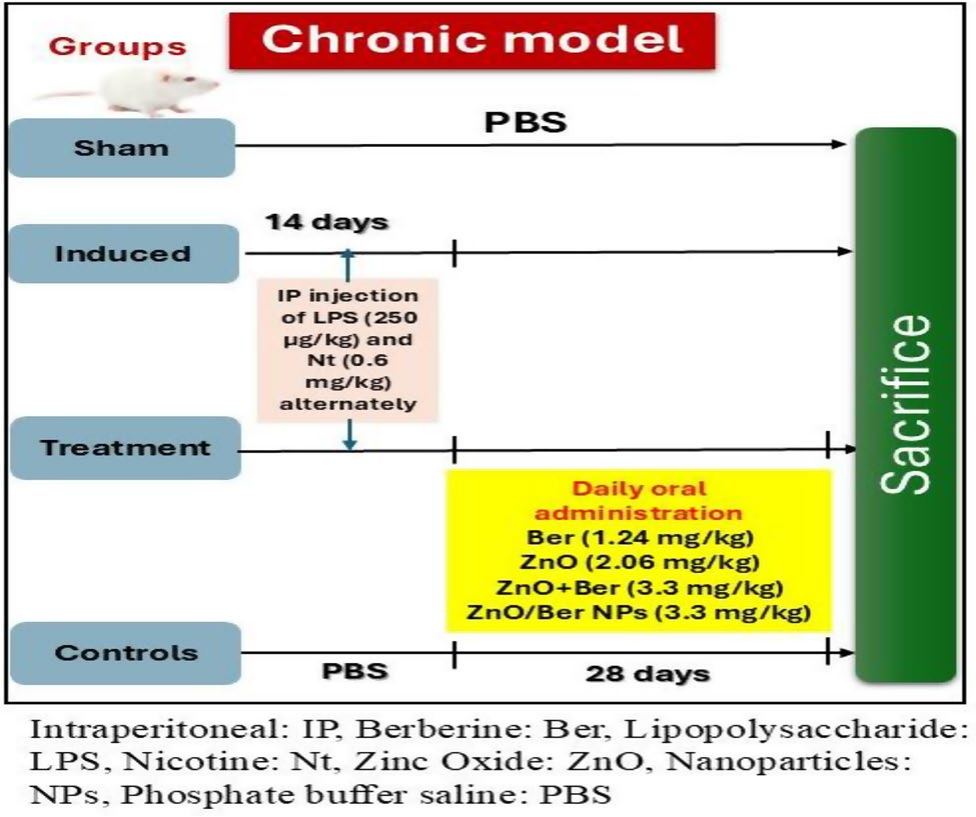
Schematic diagram of animal experimental design

The mice were separated into three main groups and a sham control group (6 mice) (Fig. 1). The induced group (LPS + Nt) (6 mice) was given alternate intraperitoneal (IP) injections of LPS (250 µg/kg) dissolved in distilled water [25] or nicotine (0.6 mg/kg) in polyethylene glycol [26] for 14 days.

The treated group (24 mice) was initially administered LPS and nicotine alternately for 14 days and then divided based on the orally administered treatment into four subgroups as follows:

##### LPS + Nt-Ber group

administered LPS and Nt then aqueous solution of Ber (1.24 mg/kg) for 28 days.

##### LPS + Nt-ZnO NPs group

administered LPS and Nt then aqueous solution of ZnO NPs (2.06 mg/kg) for 28 days.

##### LPS + Nt-ZnO NPs + Ber group

administered LPS and Nt then a combination of ZnO NPs and Ber (3.3 mg/kg) for 28 days.

##### LPS + Nt-ZnO/Ber NPs group

administered LPS and Nt then aqueous solution ZnO/Ber NPs (3.3 mg/kg) [1] for 28 days.

The third group was the control group (24 mice), which was administered PBS, intraperitoneally, for 14 days then divided into four subgroups as follows:

##### Ber group

administered PBS then aqueous solution of Ber (1.24 mg/kg) for 28 days.

##### ZnO NPs group

administered PBS then aqueous solution of ZnO NPs (2.06 mg/kg) for 28 days.

##### ZnO NPs + Ber group

administered PBS then a combination of ZnO NPs and Ber (3.3 mg/kg) for 28 days.

##### ZnO/Ber NPs group

administered PBS then aqueous solution ZnO/Ber NPs (3.3 mg/kg) for 28 days.

At the end of the experimental period, the mice were fasted overnight before being anesthetized via isoflurane inhalation (5%) for 2–3 min [27] to minimize animal suffering. Euthanasia was performed by abdominal incision followed by collecting blood samples from inferior vena cava, before being cut, in test tubes containing heparin. After that the lung tissues were harvested where, a portion of the lung tissue from each animal group was preserved in a 10% formalin solution for histological analysis, while the remaining lungs were rinsed with cold 0.9% saline and stored at -80 °C for subsequent studies.

#### Biochemical analyses

The C-reactive protein level was measured via a CRP Turbi latex kit (Spectrum Diagnostics, Egypt) following the directions provided by the manufacturer.

#### Oxidative stress assessments

The frozen lung tissues were homogenized in lysis buffer containing protease inhibitor (pH 7.4). Following homogenization, the materials were centrifuged for 10 min at 22,673 *×* g and 4 °C. The supernatant was collected, and the total protein content was subsequently quantified via the Lowry method, as described by Waterborg [28].

Lung homogenates were used to assess oxidative stress markers, inflammation, and lung lesions. The oxidative stress parameters, including glutathione-s-transferase (GST), were measured by Habig et al. [29], superoxide dismutase (SOD) was measured via the Marklund and Marklund method [30], thiobarbituric acid reactive substances (TBARS) were measured via Tappel & Zalkin [31], reduced glutathione (GSH) was determined via Ellman’s method [32], and glutathione peroxidase (GPx)-specific activity was reported by Paglia & Valentine [33].

#### Inflammatory markers assessment

In accordance with the manufacturer’s instructions, sandwich enzyme-linked immunosorbent assay (ELISA) was used to assess various inflammatory markers, such as mouse IL-10, interferon gamma (IFNγ), IL-1β, and TNFα. In addition, manual indirect ELISA was used to measure the following lung lesion markers: TREM-1, MPO, and ACE II. Indirect ELISA was performed according to the abcam protocol by manually coating antigens into a microtiter plate with 200 mM coating buffer comprising sodium carbonate and sodium bicarbonate at pH 9.6. The plate was then left for two h at room temperature and incubated overnight at 4 °C. The following day, 5% bovine serum albumin was added for blocking, and the samples were incubated for one h at 37 °C, followed by washing 3 times with phosphate-buffered saline (PBS) (pH 7.4). The wells were then incubated with the primary antibody (1:1000) for two h at room temperature. Following incubation, the unbound antibody was removed, and the wells were incubated with horseradish peroxidase (HRP)-conjugated secondary antibody (1:1000) for 1.5 h. After incubation, the unbound antibody was removed, and the TMB substrate was added and incubated for 30 min at 37 °C in the dark. The reaction was then stopped via the addition of 5% H_2_SO_4,_ after which the absorbance was measured at 450 nm.

#### Quantification of the gene expression levels of apoptosis markers and NF-kB

The gene expression levels of apoptosis markers, including Bax, p53, and NF-κB, were quantified via qRT‒PCR. RNA was isolated from mouse lungs by homogenizing the tissue with TRIzol reagent. The total RNA was subsequently extracted via chloroform and isopropanol following the manufacturer’s established process. The complementary DNA (cDNA) was subsequently generated from 5 µg of total RNA via the TOPscript RT DryMIX (dT18/dN6 plus) commercial kit manufactured by Enzynomics, Munji-ro, Yuseong-gu, Daejeon, Republic of Korea. A StepOnePlus RT‒PCR system (Thermo Fisher Scientific, Inc., Waltham, MA, USA) was used to perform real-time quantitative PCR. A TOPreal qPCR 2X PreMIX commercial kit (Enzynomics, Munji-ro, Yuseong-gu, Daejeon, Republic of Korea) was utilized, with 1 µL of cDNA used as the template. The samples were normalized to the values for the GAPDH gene. The sequences of the primers used are listed in Table 1.

**Table 1:** Real-time PCR forward and reverse primer sequences

Gene	Forward primer sequence	Reverse primer sequence	Ref.
GAPDH	5’-GGCAAATTCAACGGCACAGT-3’	5’-GTCTCGCTCCTGGAAGATGG-3’	[34]
Bax	5’-ATGTTTTCTGACGGCAACTTC-3’	5’-AGTCCAATGTCCAGCCCAT-3’	[35]
*P53*	*5’-ATGTTTTGCCAACTGGCCAAG-3’*	*5’-TGAGCAGCGCTCATGGTG-3’*	[35]
*Nf-kb*	5′-CGCAAAAGGACCTACGAGAC-3′	5′-TGGGGGAAAACTCATCAAAG-3′	[36]

#### Histopathological analysis

Lung tissue samples were fixed in 10% formaldehyde, embedded in paraffin, and sectioned into 5 μm slices via a manual rotary microtome. The sections were then stained with hematoxylin and eosin for analysis [37]. Pathologic changes were examined via light microscopy, and multiple images were captured at ×100 magnification via a Leica digital camera. A pathologist who was blinded to the slides evaluated the images. For the chronic lung injury score, the extent of the pathological lesions was graded from 0 to 3 with respect to perivascular inflammatory infiltrate with parenchymal destruction, peribronchial inflammatory infiltrate with parenchymal destruction, and alveolar inflammatory infiltrate with compressed alveoli per power field. At least five fields were assessed, and the mean was taken. The score for each animal was calculated by dividing the total score by the number of sections observed.

#### In silico analysis

##### Ligand and protein preparation

ZnO/Ber, berberine and ZnO were subjected to active site molecular docking to investigate their bioactivities toward five selected proteins. The 3D conformers of berberine and ZnO were downloaded in SDF file formats from https://pubchem.ncbi.nlm.nih.gov. ZnO/Ber was designed in PDB file format on the Avogadro program, which is also used to optimize its energy. Open Babel software was used to convert the ligand files into PDBQT files prior to molecular docking simulations. Five selected proteins were obtained from https://www.rcsb.org. Swiss-PdbViewer software was used to process these receptors’ energy optimization procedures and determine whether any protein segments were missing. The proteins were created by eliminating all heteroatoms and solvent molecules via Discovery Studio Visualizer Software, version 4.0. The active sites of the chosen proteins were discovered via Discovery Studio Visualizer Software, which was then utilized to construct the grid box for the molecular docking simulation. Ligplot + software suggested possible hydrophobic interactions between the studied ligands and the different amino acids of the examined proteins.

##### Molecular docking parameters

Following the preparation of drug-like compounds and selected proteins and the optimization of their energies to represent their natural stability, the compounds were subjected to an active site molecular docking method, as this simulation is contingent upon energy considerations. The molecular docking simulation was performed via Autodock 4 (Autodock tool, version 1.5.6rc3). Polar hydrogens were added to the chosen proteins at the start of the procedure, Kollman charges were then integrated, and Gasteiger charges were assigned. The grid boxes were designed to cover the active site areas to generate maps and to facilitate the expression of the resulting noncovalent interactions (NCIs). The grid parameters were set to 60 × 60 × 60 points with a spacing of 0.375 Å. The coordinates of the active sites were as follows: (x = 20.634, y= -2.685, z= -16.848) for the 2R37 receptor and (x = 20.170, y = 34.284, z = 16.608) for the 1PL4 receptor, whereas (x = 17.752, y = 38.682, z = 16.771). On the other hand, (x = 20.593, y = 100.916, z = 10.681) for the 1Q8M receptor, and (x= -23.671, y= -6.537, z = 31.829) for the 6WYZ receptor. The docking parameters were adjusted to genetic algorithms, 50 conformations were identified, and the optimal conformer was chosen on the basis of the lowest free energy of binding and the minimal inhibition constant, Ki. The resulting ligand–protein complexes were then investigated for their hydrophilicity via Ligplot + software. PyMol was used for visualization.

##### In silico pharmacokinetics properties

The potential protein targets and pharmacokinetic properties of Ber and ZnO/Ber were predicted by Swiss Target Prediction (https://www.swisstargetprediction.ch/) and SwissADME (https://www.swissadme.ch/) tools provided by Swiss Bioinformatics Institute. Swiss Target Prediction predicts the most probable macromolecular targets of small molecules based on a combination of 2D and 3D similarity measures with known ligands from the ChEMBL database. Swiss ADME calculates ADME parameters: Absorption, Distribution, Metabolism, and Excretion.

#### Statistical analysis

Statistical analysis was conducted via one-way ANOVA followed by Tukey’s post hoc test in SPSS version 22.0. The data are presented as the means ± SDs, with significance determined at *p* < 0.05.

## Results

### Physicochemical characterization of ZnO/Ber NPs

Characterization of the ZnO-Ber NP complex FT-IR spectra of ZnO and the ZnO/Ber NPs was performed via a Shimadzu IRTracer-100 FT-IR spectrophotometer. in KBr disks, in the wavenumber range of 400–4000 cm^− 1^.

**Fig. 2:**
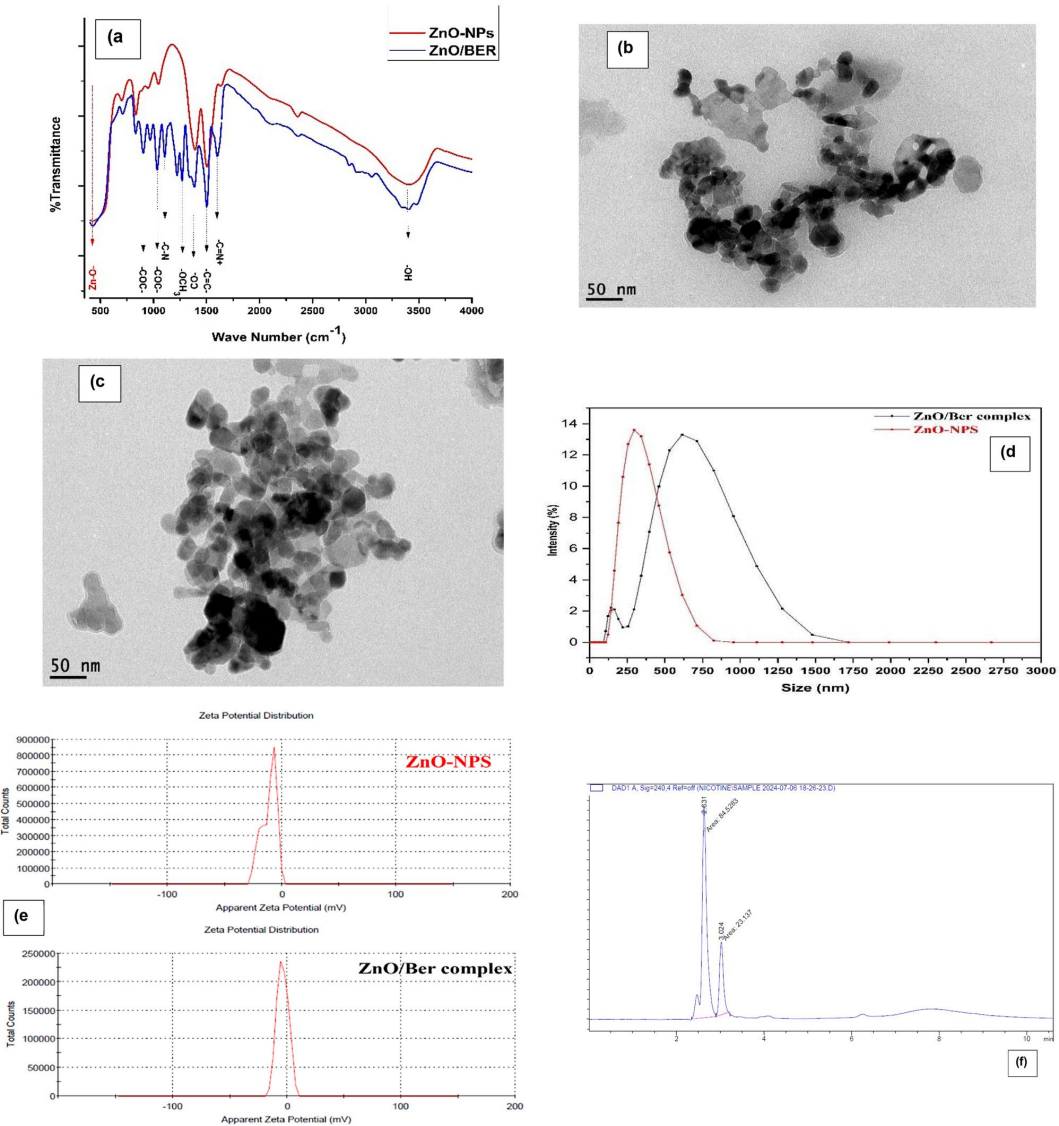
FT-IR spectral analysis (**a**), TEM image of ZnO NPs (**b**) and ZnO/Ber NPs (**c**), hydrodynamic size (nm) of ZnO-NPs (335 nm) and ZnO/BER -NPs (551 nm) aqueous suspensions (**d**), zeta potential in (mv) of ZnO-NPs (− 11 mv) and ZnO/BER complex (− 3.77 mv) aqueous suspensions (**e**), and chromatogram of commercial nicotine (**f**)

The FT-IR spectra (Fig. 2a) revealed the existence of a pair of CH_2_-O-CH_2_ ether group peaks at Ber (904 and 1034) cm^− 1^_,_ a Ber–OCH3 methoxy group peak (1104, 2846) cm^− 1^, a Ber iminium peak (C = N+), a characteristic sharp peak at 1598 cm^− 1^, and a -C-N vibration peak at 1164 and 1567 cm^− 1^.

In the FTIR spectra of ZnO and ZnO-Ber, prominent vibration bands corresponding to the characteristic stretching mode of the Zn‒O bond were observed at 427.25 cm⁻¹ and 428.21 cm⁻¹, respectively, for both compounds. A broad and weak peak at 3403–3407 cm⁻¹ (stretching) along with peaks between 1391 cm⁻¹ and 1632 cm⁻¹ (bending) suggest the presence of hydroxyl residues, which are likely attributed to atmospheric moisture and hydroxyl groups on the surface of the ZnO particles.

The FT-IR spectra of the ZnO/Ber NPs verify the interactions between the principal functional groups of Ber and ZnO and confirm the successful preparation of the nanocomplex.

TEM analysis was conducted to examine the size and morphology of the ZnO/Ber NPs via a JEOL JEM 1400 instrument. TEM images (Fig. 2b and c) revealed ZnO/Berberine agglomerated clusters from ZnO Nps shielded by a light organic layer of berberine with a particle size of 57 ± 9 nm.

The dynamic light scattering (DLS) technique using Zetasizer Nano ZS (Malvern, Worcestershire, UK) instrument was used to measure the hydrodynamic size, polydispersity index, and zeta potential of free ZnO NPs and ZnO/Ber complex dispersed in milliQ water.

Figure 2d and e show that the average hydrodynamic size of ZnO NPs was about 335 ± 123 nm due to their tendency to aggregate in aqueous suspension, with a polydispersity index (PDI) of 0.331 and zeta potential equals − 11 mV, whereas the average hydrodynamic size of ZnO/Ber complex was about 551 ± 243.5 nm, with PDI of 0.356 and zeta potential equals − 3.77 mV. This increase in size and higher aggregation is attributed to the electrostatic interaction between the anionic ZnO NPs and the cationic berberine molecules, which reduces the complex’s surface potential and thereby promotes greater aggregation. This deals completely with our group’s previous work at Ghareeb et al. [1].

### Nicotine concentration in commercial nicotine

The chromatogram of commercial nicotine showed peaks at retention times of 2.631 and 3.024, indicating that the concentration of nicotine in the sample was 43.47 ng/µL (Fig. 2f).

### Oxidative stress parameter levels during induction and treatments

**Fig. 3:**
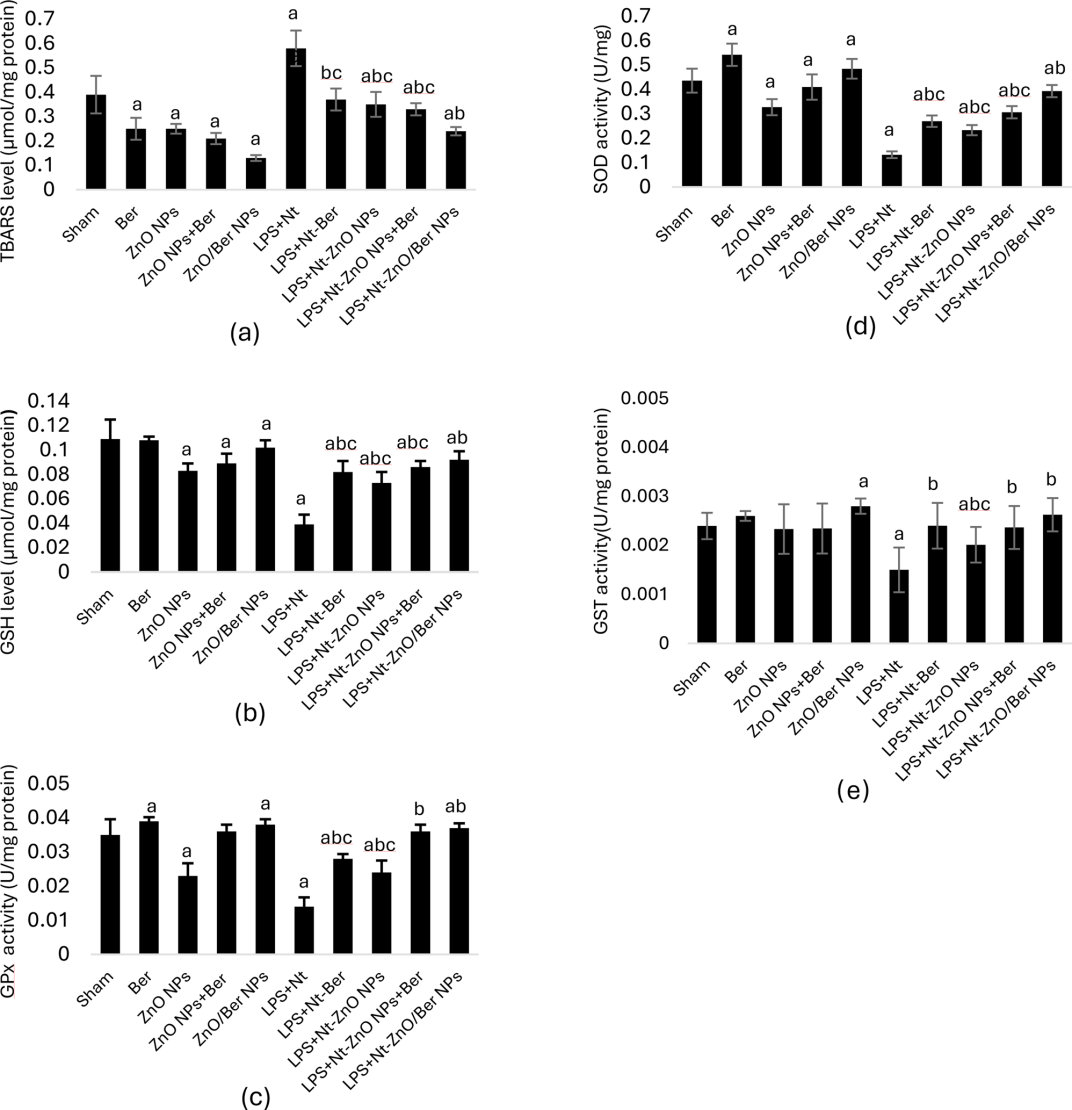
Levels of oxidative stress parameters in lung homogenates. Level of TBARS (**a**). Level of GSH (**b**). Activity of GPx (**c**). Activity of SOD (**d**). Activity of GST (**e**). Data are expressed as mean ± standard deviation, bars with **a** are statistically significant at *p* < 0.05 compared to sham control group, bars with **b** are statistically significant at *p* < 0.05 compared to LPS + Nt induction group, bars with **c** are statistically significant at *p* < 0.05 compared to LPS + Nt-ZnO/Ber NPs treatment group

Compared with the sham group, chronic induction with LPS + Nt led to a significant increase in the level of TBARS (*p* < 0.05). Compared with the sham group, the 28-day treatment groups had significantly lower TBARS levels (*p* < 0.05). All treatments administrated to induced mice significantly decreased TBARS levels compared to LPS + Nt induction and sham groups (*p* < 0.05). Notably, the treatment with ZnO/Ber NPs resulted in the lowest TBARS level compared with that of the other therapies, with ~ 60% decrease compared with induction group at *p* < 0.05 (Fig. 3a).

After 28 days, GSH levels in both healthy and induced mice were lower than sham, except for the Ber-treated healthy group, which matched sham level (*p* < 0.05). Chronic LPS + Nt induction significantly reduced GSH (*p* < 0.05), but treatment with Ber, ZnO NPs, ZnO NPs + Ber, and ZnO/Ber NPs increased levels by 110%, 87%, 120%, and 135%, respectively, compared to induction (*p* < 0.05) (Fig. 3b).

Chronic LPS + Nt induction, as well as the administration of ZnO NPs to healthy mice, and Ber or ZnO NPs alone to induced mice, led to a significant decrease in GPx activity, compared with sham group (*p* < 0.05). However, treatments significantly boosted GPx activity relative to the induction group, with ZnO/Ber NPs showing the most pronounced effect with 164% increase in GPx activity (*p* < 0.05) (Fig. 3c).

LPS + Nt induction also significantly lowered SOD enzyme activity (*p* < 0.05). Compared with the sham control, ZnO NPs and ZnO NPs + Ber reduced SOD activity in healthy mice, whereas Ber and ZnO/Ber NPs increased it, significantly (*p* < 0.05). Among the treatments, Ber significantly enhanced enzyme activity, whereas ZnO NPs showed significant reduction (*p* < 0.05). Similarly, compared with sham control, LPS + Nt induction reduced GST activity. Healthy mice treated for 28 days maintained sham GST levels, except those receiving ZnO/Ber NPs, whose levels significantly increased (*p* < 0.05). Compared with induction alone, postinduction treatments also significantly elevated GST activity (*p* < 0.05) (Fig. 3d-e).

### Inflammatory markers and serum C-reactive protein levels during induction and treatments

Compared with the sham control, LPS + Nt significantly increased the IL-10, TNF-α, and IFN-ɣ levels by ~ two-folds but had no effect on the IL-1β concentration. Compared with sham control mice, healthy mice subjected to different treatments for 28 days presented normal concentrations of TNF-α, IFN-ɣ and IL-1β but significantly lower IL-10 concentrations, with the exception of ZnO NPs, which presented normal IL-10 levels (*p* < 0.05).

**Fig. 4:**
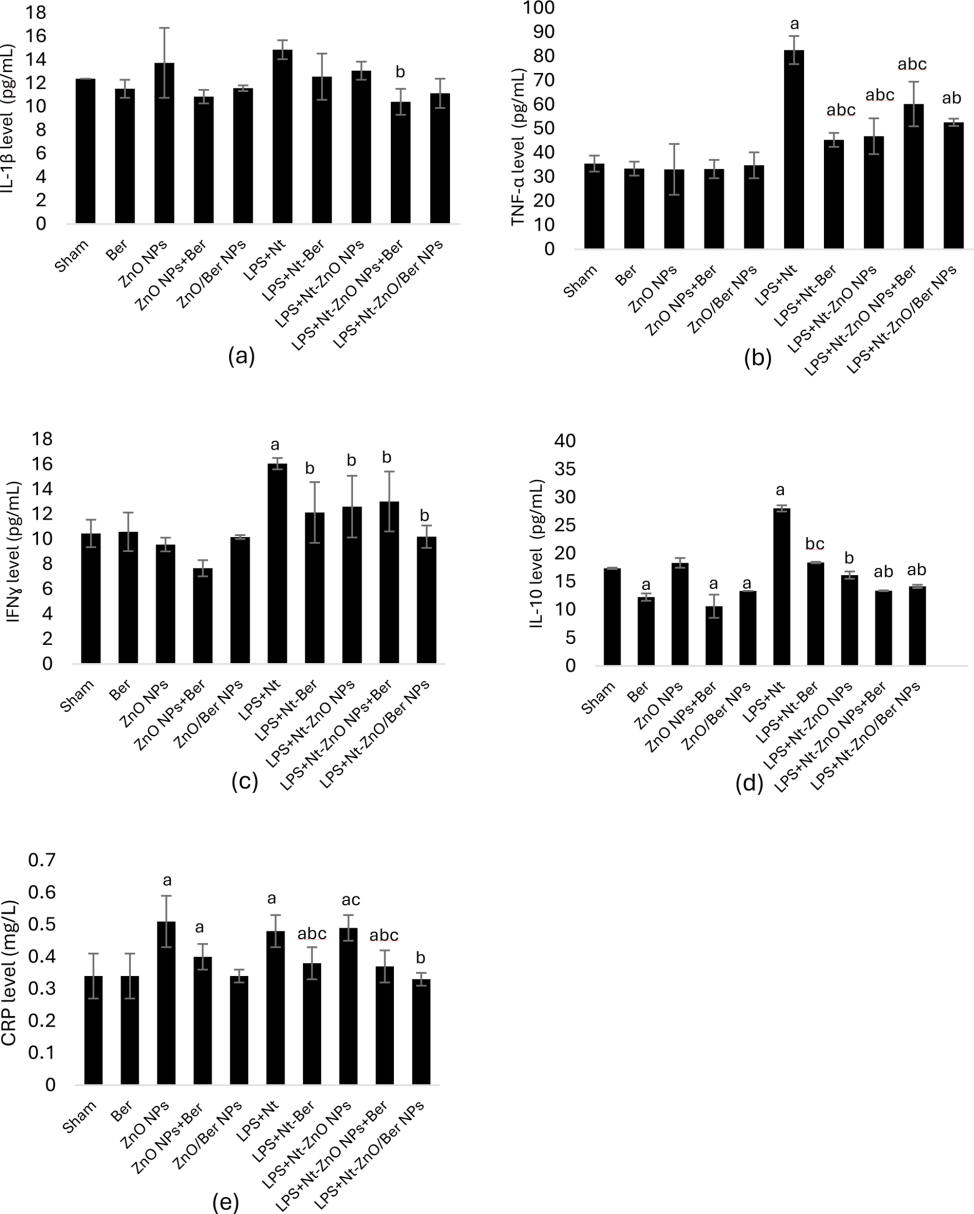
Level of inflammatory markers in lung homogenate. Data are expressed as mean ± standard deviation, bars with **a** are statistically significant at *p* < 0.05 compared to sham control group, bars with **b** are statistically significant at *p* < 0.05 compared to LPS + Nt induction group, bars with **c** are statistically significant at *p* < 0.05 compared to LPS + Nt-ZnO/Ber NPs treatment group

ARDS-induced mice administered different treatments for four weeks showed significantly lower IL-10, IFNɣ and TNF- α concentrations than induction group (*p* < 0.05). Compared to the induction group, the ZnO NPs + Ber mixture and ZnO/Ber NPs significantly decreased IL-1β concentration with no difference in Ber and ZnO NPs groups at *p* < 0.05 (Fig. 4a-d).

CRP was investigated in the serum of the examined groups as a crucial indicator of inflammation. LPS + Nt induction caused a significant increase in the CRP level at *p* < 0.05. Healthy mice that were administered Ber or ZnO/Ber NPs for 28 days showed the same control level, whereas those that were administered ZnO NPs or the ZnO NP + Ber mixture presented higher CRP levels than the sham control (*p* < 0.05). Treatment with ZnO NPs alone did not significantly reduce CRP levels at *p* < 0.05. However, both Ber and ZnO NPs + Ber decreased CRP value, and ZnO/Ber NPs had the most substantial effect (~ 35% reduction than LPS + Nt group) (*p* < 0.05) (Fig. 4e).

### Lung lesion marker levels during induction and treatment

**Fig. 5:**
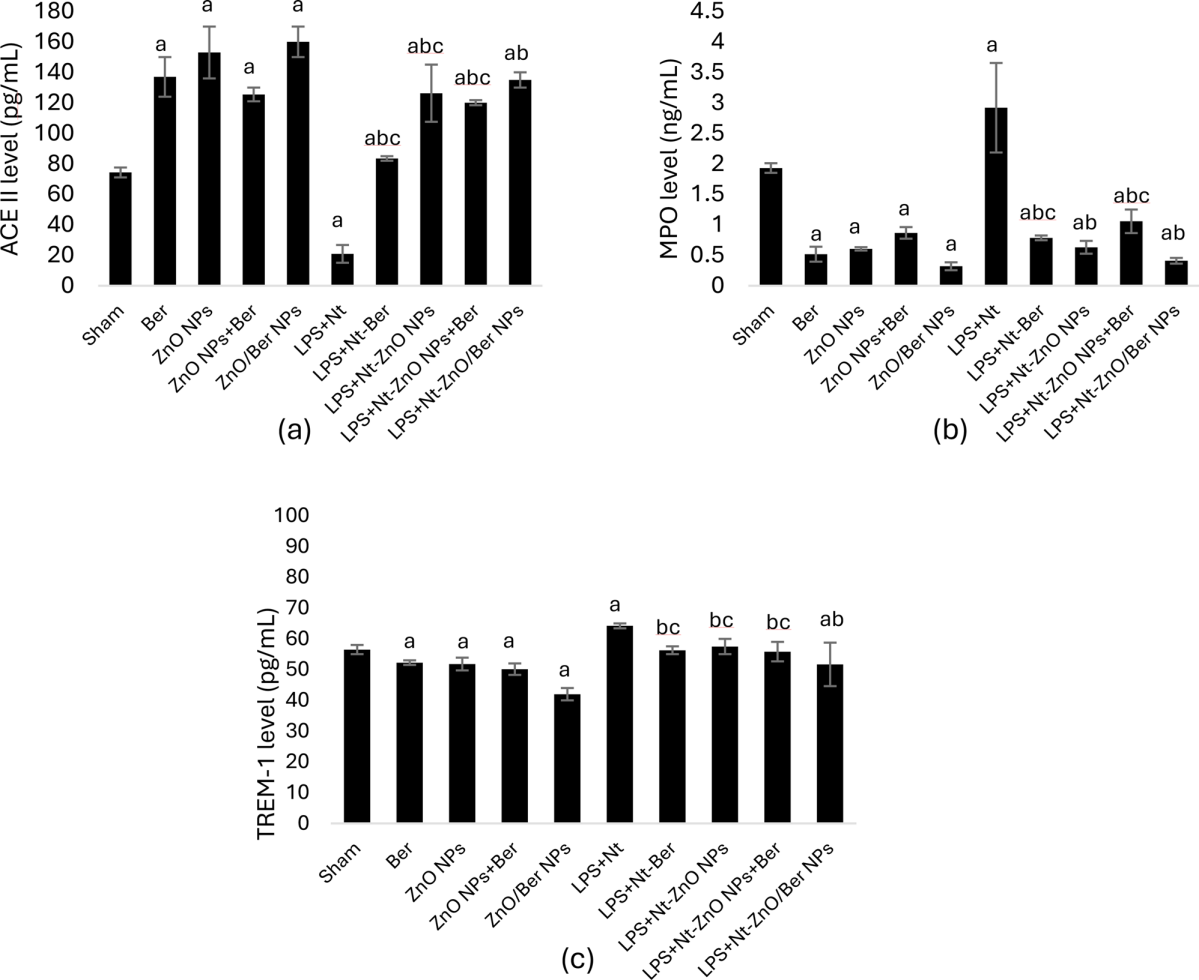
Level of lung lesion markers in lung homogenate. Data are expressed as mean ± standard deviation, bars with a are statistically significant at *p* < 0.05 compared to shamcontrol group, bars with b are statistically significant at *p* < 0.05 compared to LPS + Nt induction group, bars with c are statistically significant at *p* < 0.05 compared to LPS + Nt-ZnO/Ber NPs treatment group

Figure 5 shows that ARDS induction significantly decreased ACE II levels with 71% and increased MPO and TREM-1 levels compared with those of the sham control at *p* < 0.05. Compared with those in the sham control group, the concentration of ACE II increased with different treatments administered for 28 days, whereas the levels of MPO and TREM-1 decreased (*p* < 0.05).

The administration of different treatments four weeks after chronic induction significantly reduced MPO and TREM-1 levels but replenished ACE II compared with those in the induction group (*p* < 0.05). ZnO/Ber NPs exhibited a superior effect over the other treatments, with 6-fold increase in ACE II concentration while, 7-fold and one and half-fold decrease in MPO and TREM-1 concentrations, respectively (*p* < 0.05).

### Apoptotic gene expression levels during induction and treatment

Table 2 shows that chronic ARDS induction significantly increased Bax, p53, and NF-κB expression levels at *p* < 0.05. Compared with those in the sham control group, healthy mice that received different treatments presented normal expression levels of the three genes. Compared with those in the induction group, the expression levels of genes in the induced group subjected to different treatments were significantly lower (*p* < 0.05). Compared with the other treatments, ZnO/Ber NPs presented the lowest expression levels at *p* < 0.05, with the exception that there is no significant difference between ZnO/Ber NPs and ZnO NPs + Ber in Bax and NF-κB expression levels (*p* < 0.05).

**Table 2:** Expression levels of apoptotic genes in lung tissue

	Bax	NF-κB	P53
Sham	1.06 ± 0.135	1.12 ± 0.212	1.08 ± 0.115
Ber	1.53 ± 0.195	1.24 ± 0.158	1.12 ± 0.143
ZnO NPs	0.98 ± 0.125	1.18 ± 0.150	1.08 ± 0.138
ZnO NPs + Ber	1.03 ± 0.131	1.19 ± 0.152	1.09 ± 0.139
ZnO/Ber NPs	1.09 ± 0.139	1.29 ± 0.164	1.01 ± 0.129
LPS + Nt	436.14 ± 52.243^a^	344.29 ± 49.619^a^	368.29 ± 49.362^a^
LPS + Nt-Ber	96 ± 1.521^abc^	163 ± 0.196^abc^	116 ± 0.196^abc^
LPS + Nt-ZnO NPs	177 ± 0.691^abc^	191 ± 0.827^abc^	180 ± 0.847^abc^
LPS + Nt-ZnO NPs + Ber	39.1 ± 0.469^b^	95 ± 1.138^ab^	102 ± 1.139^abc^
LPS + Nt-ZnO/Ber NPs	19.7 ± 1.146^b^	55.66 ± 6.668^ab^	50.93 ± 6.655^ab^

The data are expressed as the mean ± standard deviation; means with **a** are statistically significant at *p* < 0.05 compared with the sham control group; means with **b** are statistically significant at *p* < 0.05 compared with the LPS+Nt induction group; means with **c** are statistically significant at *p* < 0.05 compared with the LPS+Nt−ZnO/Ber NP treatment group

### Histopathology of lung tissues during induction and treatment

The histological assessment of lung sections was performed after 42 days, which was at the end of the experiment.

**Fig. 6:**
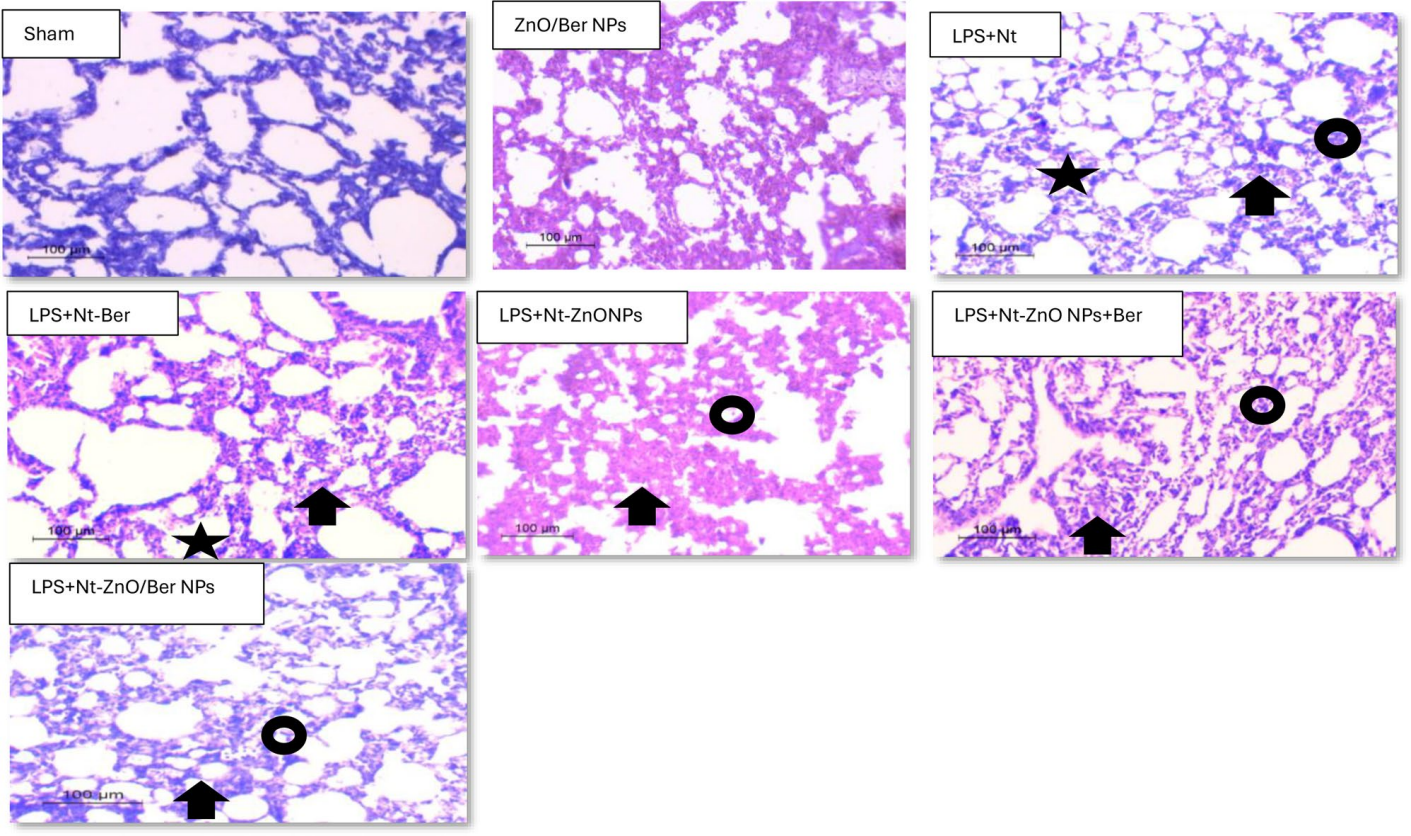
Histopathological changes and lung injury score of chronic model (x20), scale bar = 100 microns. **(**sham): showed normal alveoli within average wall thickness, no inflammation. H&E (x20), (ZnO/Ber NPs): showed normal alveoli within average wall thickness, no inflammation.H&E (x20), (LPs + NT): The alveoli showed marked alveolar (arrow)and peribronchial inflammatory infltrate (circle) with parenchymal destruction (star) H&E (x20), median score 3, (LPS + Nt-Ber): The alveoli showed moderate alveolar inflammatory infiltrate (arrow) with parenchymal destruction (star) H&E (x20), median score 2, (LPS + Nt-ZnO NPs): The alveoli showed moderate alveolar inflammatory infiltrate (arrow) with parenchymal destruction (star) H&E (x20), median score 2, (LPS + Nt-ZnO NPs + Ber): The alveoli showed moderate alveolar inflammatory infiltrate (arrow) with parenchymal destruction (star) H&E (x20), median score 2 (LPS + Nt-ZnO/Ber NPs): The alveoli showed mild alveolar inflammatory infiltrate (arrow) with parenchymal destruction (star) H&E (x20), median score 1

LPS + Nt induction for 14 days resulted in marked perivascular inflammatory infiltration, peribronchial inflammatory infiltration, parenchymal destruction, and alveolar inflammatory infiltration with compressed alveoli. Administration of different treatments effectively reduced airspace inflammation, which was more apparent in the mice treated with the ZnO/Ber NPs than in those treated with the other compounds. Moreover, the severity of lung injury was scored via a semiquantitative histopathology scoring system, which evaluates lung injury in four categories: perivascular inflammatory infiltrate with parenchymal destruction, peribronchial inflammatory infiltrate with parenchymal destruction and alveolar inflammatory infiltrate with compressed alveoli. We found that treatment with ZnO/Ber NPs significantly reduced the lung injury score, which was more apparent in the mice treated with ZnO/Ber NPs than in those treated with the ZnO NP, Ber, or ZnO NPs + Ber mixture (Fig. 6). Notably, similar to the sham control, ZnO/Ber NPs had no adverse effects on healthy tissue with normal alveoli and no inflammation.

### Molecular docking simulation

The bioactivities of the ZnO/Ber complex and its parent, berberine and ZnO, were examined against the following proteins: GPx, code 2R37; SOD protein, code 1PL4; ACE2 protein, code 6M1D; TREM1 protein, code 1Q8M; and MPO protein, code 6WYZ.

Table 3 shows that together with the results of in vivo tests, the results of in silico analyses confirmed that ZnO/berberine had the strongest GPx binding, with a free energy of -12.19 kcal/mol and an inhibition constant of 1.15 nM, whereas ZnO had the weakest interaction (-2.48 kcal/mol, Ki = 15.18 mM). Hydrogen bonding was observed for ZnO/berberine (GLU136, PRO100) and ZnO (GLN146). In the case of SOD, ZnO/berberine had a free energy of -9.63 kcal/mol and a Ki of 86.9 nM, whereas ZnO exhibited the weakest binding (-2.88 kcal/mol, Ki = 7.75 mM). Hydrogen bonding was detected for all three compounds, with ZnO/berberine binding to GLN143.

**Table 3:** Docking results for the best conformers of the tested ligands, ZnO/berberine, Berberine and ZnO, against the studied receptors, 2R37, 1PL4, 6M1D, 1Q8M and 6WYZ

Parameter	GPx protein, code: 2R37		
	ZnO/berberine	Berberine	ZnO
Free energy of binding (Kcal/mol)	-12.19	-7.8	-2.48
Inhibition constant Ki	1.15 nM	1.91 µM	15.18 mM
Ref. RMSD	18.61	20.67	27.1
Hydrogen bonding	LIGO: GLU136(H), 2.5 A^o^.	None.	LIGO: GLN146(H), 2.2 A^o^. LIGO: GLU136(H), 2.9 A^o^. LIGO: PRO100(H), 2.9 A^o^.
Parameter	SOD protein, code: 1PL4		
	ZnO/berberine	Berberine	ZnO
Free energy of binding (Kcal/mol)	-9.63	-6.74	-2.88
Inhibition constant Ki	86.9 nM	11.4 µM	7.75 mM
Ref. RMSD	39.52	42.7	48.3
Hydrogen bonding	LIGO: GLN143(H), 2.0 A^o^.	LIGO: GLN143(H), 2.2 A^o^.	LIGO: ASN142(H), 2.0, 2.7 A^o^.
Parameter	ACE2 protein, code: 6M1D		
	ZnO/berberine	Berberine	ZnO
Free energy of binding (Kcal/mol)	-9.16	-3.95	-2.64
Inhibition constant Ki	192.14 nM	1.27 mM	11.66 mM
Ref. RMSD	39.12	53.44	47.53
Hydrogen bonding	LIGO: GLN143(H), 2.1 A^o^.	LIGO: GLN119(H), 2.0 A^o^.	LIGO: ASN142(H), 2.1, 2.5 A^o^. LIGO: SER121(H), 2.1 A^o^.
Parameter	TREM1 protein, code: 1Q8M		
	ZnO/berberine	Berberine	ZnO
Free energy of binding (Kcal/mol)	-6.7	-4.68	-2.79
Inhibition constant Ki	12.31 µM	372.54 µM	9.06 mM
Ref. RMSD	100.7	104.87	115.6
Hydrogen bonding	LIGO: ASP60(H), 2.8 A^o^.	None.	LIGO: GLN117(H), 2.0, 3.4 A^o^. LIGO: GLU62(H), 3.2 A^o^.
Parameter	MPO protein, code: 6WYZ		
	ZnO/berberine	Berberine	ZnO
Free energy of binding (Kcal/mol)	-10.57	-7.67	-2.75
Inhibition constant Ki	17.72 nM	2.38 µM	9.66 mM
Ref. RMSD	49.82	55.74	41.89
Hydrogen bonding	LIGO: GLU242(H), 2.6 A^o^.	LIGO: ARG239(H), 2.2 A^o^.	LIGO: ASN165(H), 2.0 A^o^. LIGO: ILE324(H), 2.0 A^o^.

**Fig. 7:**
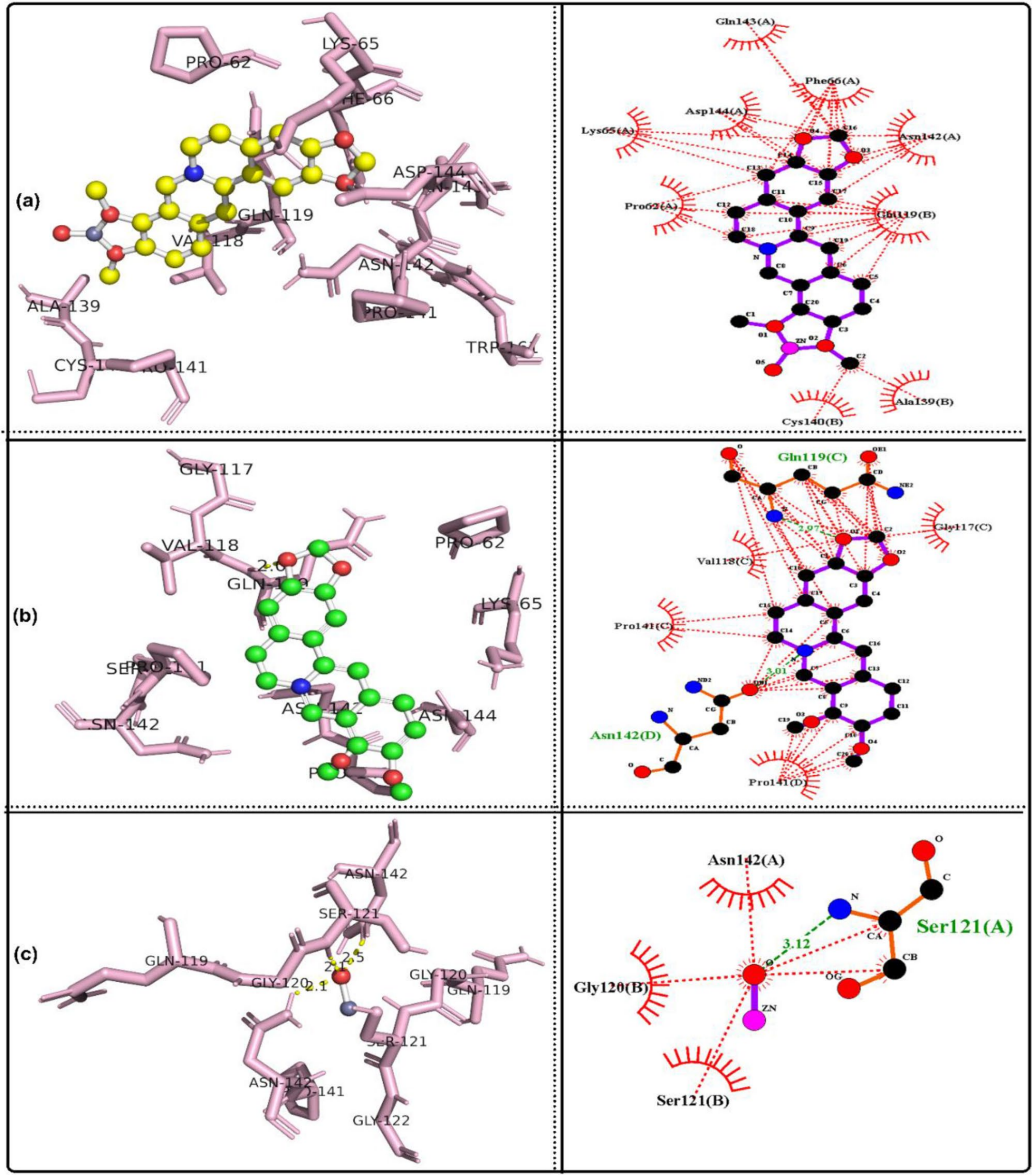
3D visualization of the best docked pose and the hydrophobic interactions of **a**: ZnO/berberine, **b**: berberine and c: ZnO against ACE2 protein, code: 6M1D

**Fig. 8:**
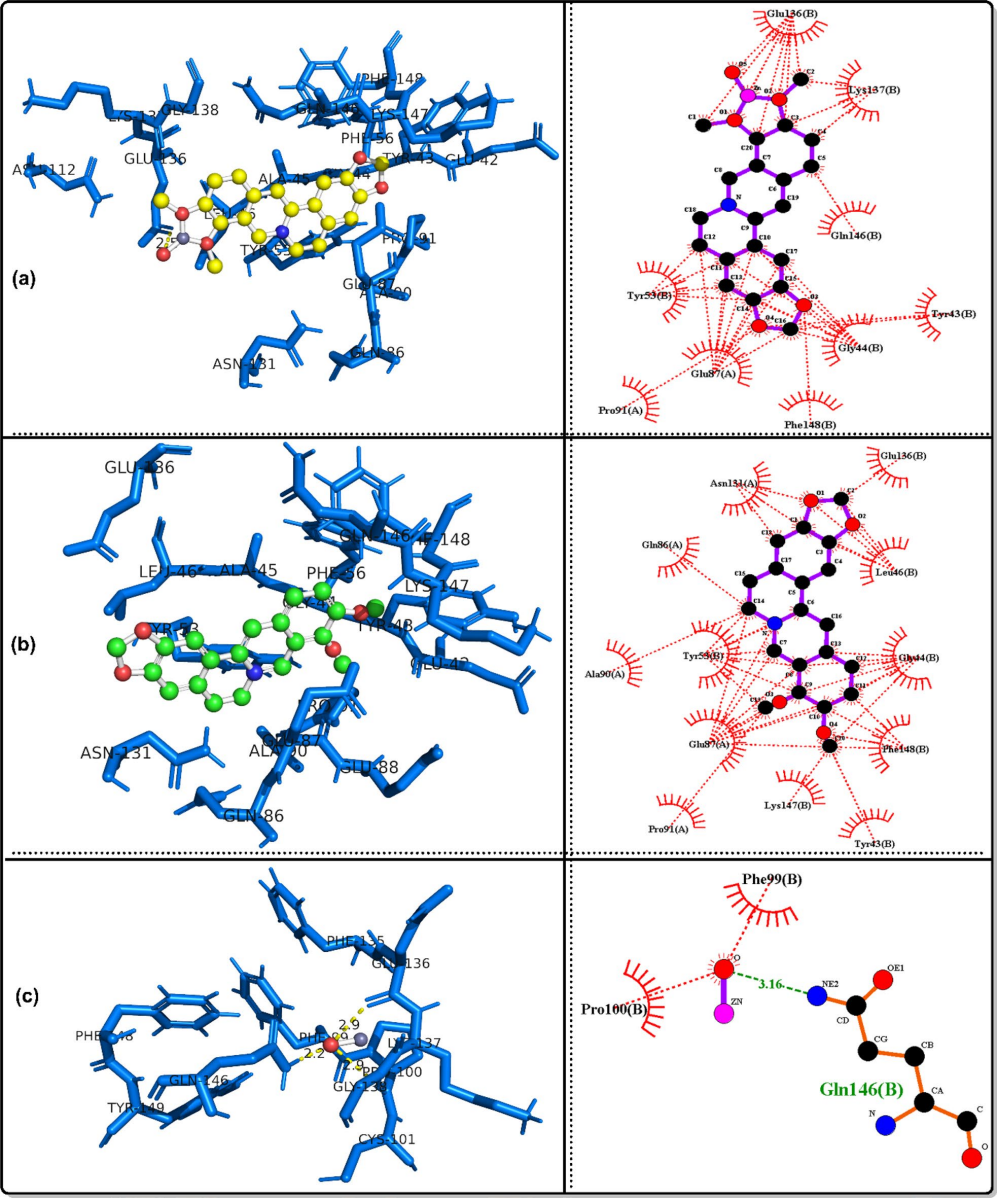
3D visualization of the best docked pose and the hydrophobic interactions of **a**: ZnO/berberine, **b**: berberine and c: ZnO against GPx protein, code: 2R37

**Fig. 9:**
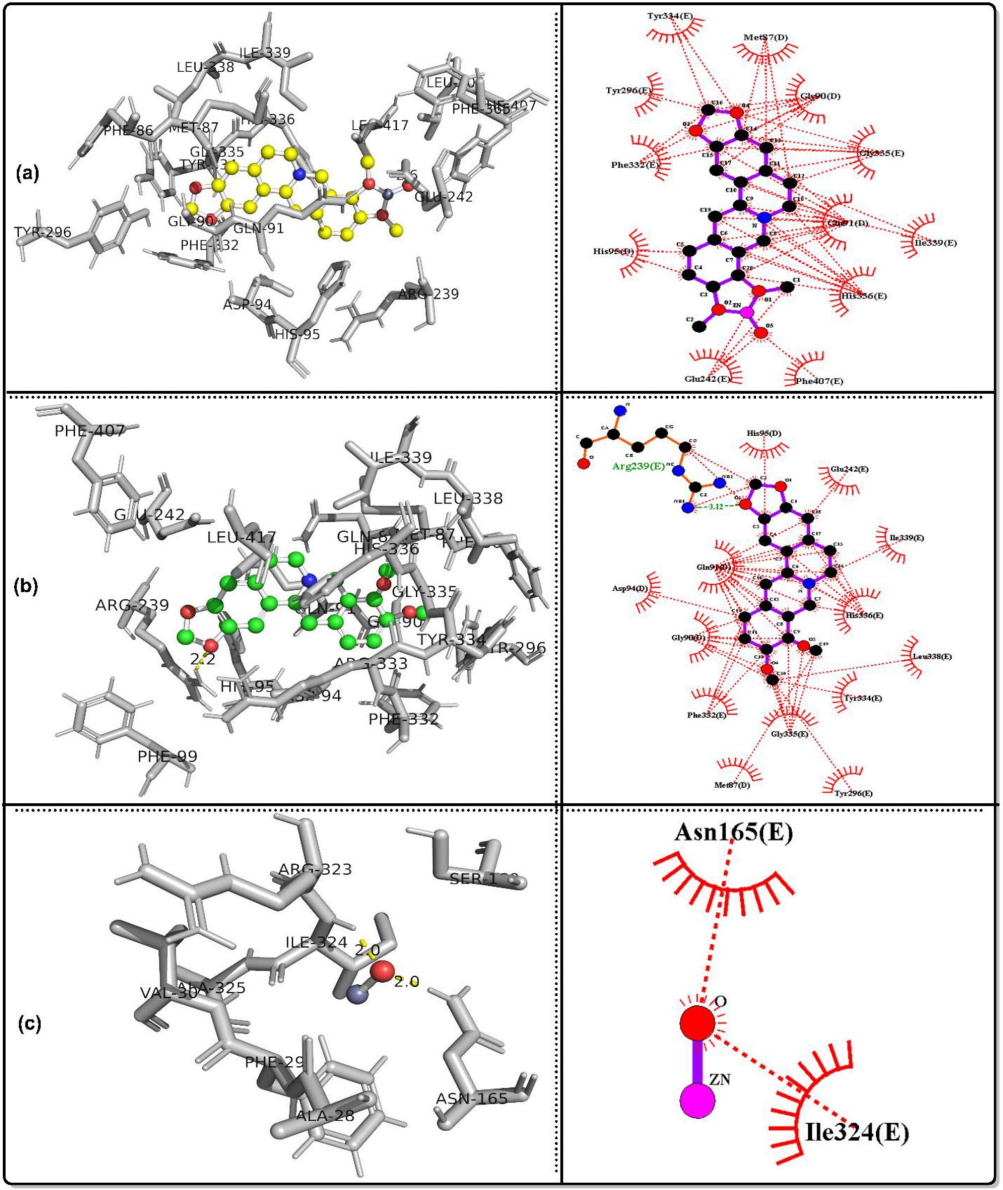
3D visualization of the best docked pose and the hydrophobic interactions of **a**: ZnO/berberine, **b**: berberine and c: ZnO against MPO protein, code: 6WYZ

**Fig. 10:**
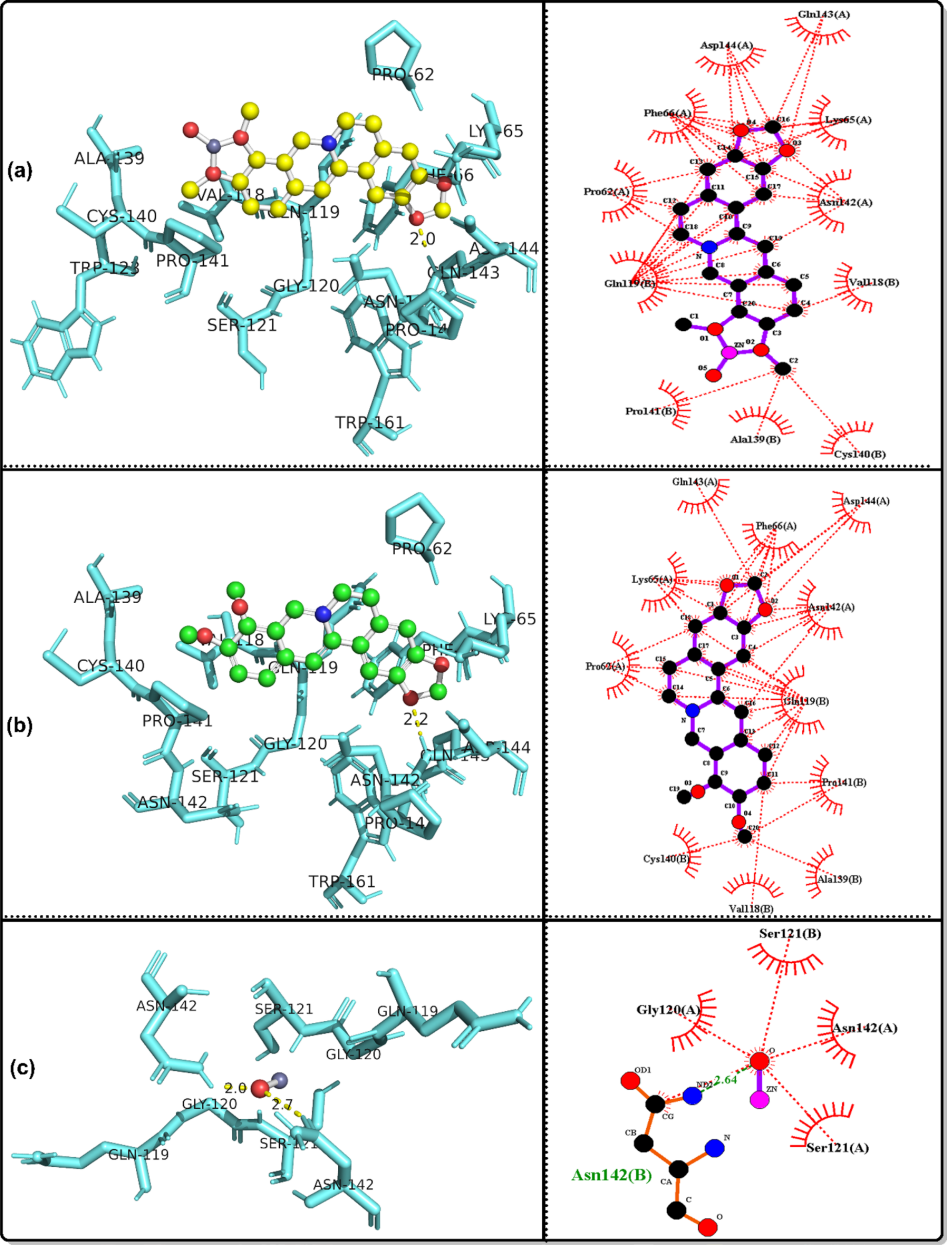
3D visualization of the best docked pose and the hydrophobic interactions of **a**: ZnO/berberine, **b**: berberine and c: ZnO against SOD protein, code: 1PL4

**Fig. 11:**
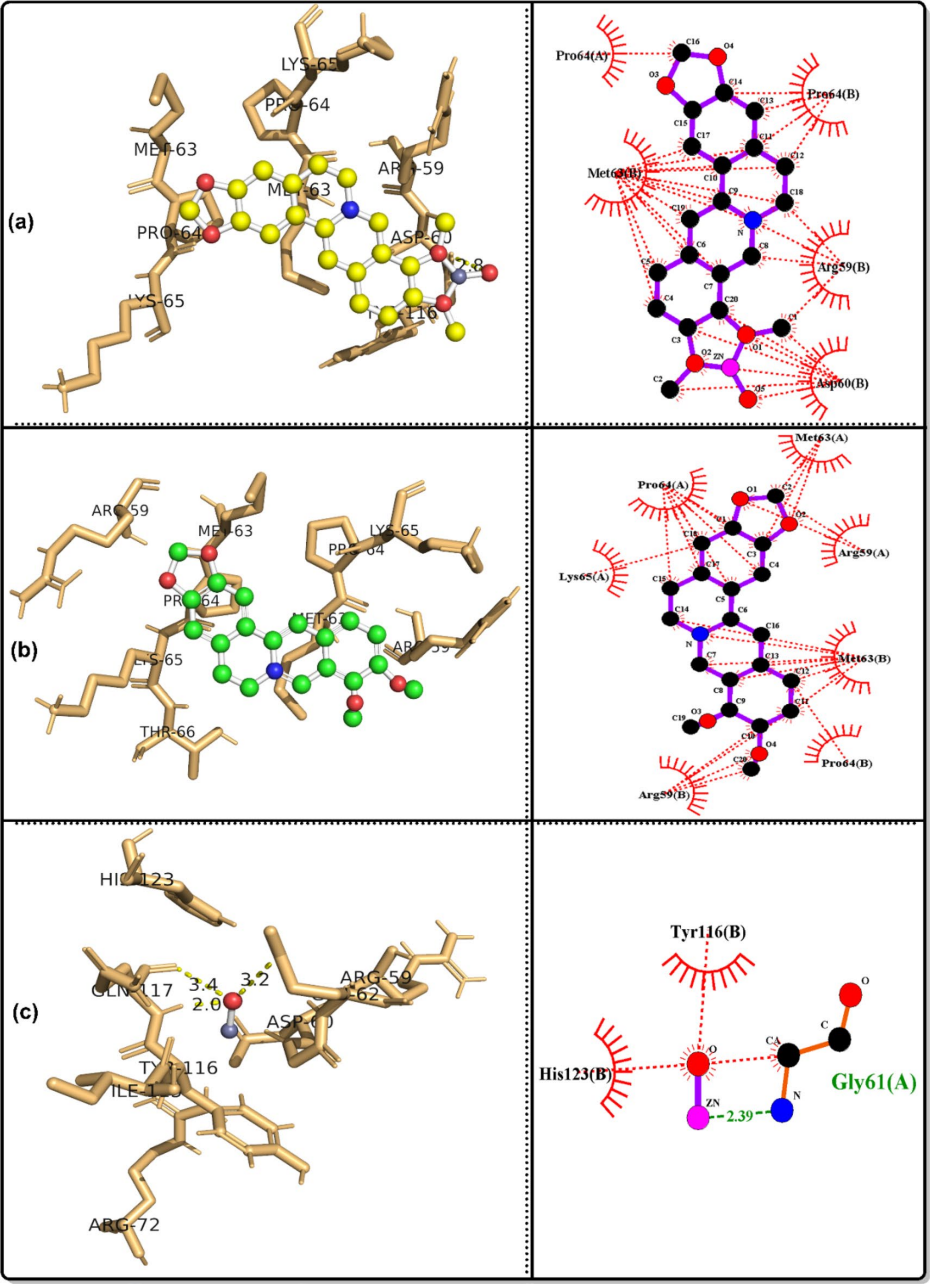
3D visualization of the best docked pose and the hydrophobic interactions of **a**: ZnO/berberine, **b**: berberine and c: ZnO against TREM1, code: 1Q8M

ZnO/berberine had a stronger affinity for ACE2 (-9.16 kcal/mol, Ki = 192.14 nM) than did berberine (-3.95 kcal/mol, Ki = 1.27 mM) and ZnO (-2.64 kcal/mol, Ki = 11.66 mM). Multiple hydrogen bonds were identified, particularly in the interactions between ZnO and ZnO/berberine. ZnO/berberine (-6.7 kcal/mol, Ki = 12.31 µM) had stronger binding with TREM1 than did ZnO (-2.79 kcal/mol, Ki = 9.06 mM). Hydrogen bonding was absent in berberine but was observed in ZnO/berberine (ASP60) and ZnO (GLN117, GLU62). Finally, ZnO/berberine had the highest affinity (-10.57 kcal/mol, Ki = 17.72 nM) for MPO, whereas ZnO had the lowest affinity (-2.75 kcal/mol, Ki = 9.66 mM). Hydrogen bonds were found in all interactions, including those of ZnO/Berberine (GLU242) and ZnO (ASN165, ILE324). Figures 7, 8, 9, 10 and 11 show 3D images of the best poses of the tested compounds with the selected receptors as well as the possible hydrophobic interactions with different receptors.

Table 4 shows that Ber and ZnO/Ber NPs show good oral absorption and blood brain barrier (BBB) permeability, suitable for systemic and CNS effects. ZnO/Ber complex has slightly better solubility but higher synthetic complexity. Both inhibit key CYP enzymes, which may affect drug metabolism and interactions.

**Table 4:** In silico ADME study

Property	Berberine	ZnO/Ber NPs
Molecular Weight (MW)	336.36 g/mol	417.74 g/mol
GI Absorption	High	High
BBB Permeant	Yes	Yes
P-gp Substrate	Yes	Yes
CYP1A2 Inhibitor	Yes	Yes
CYP2C19 Inhibitor	No	No
CYP2C9 Inhibitor	No	No
CYP2D6 Inhibitor	Yes	Yes
CYP3A4 Inhibitor	Yes	Yes
ESOL Solubility (mg/ml)	0.00953 (Moderately soluble)	0.0159 (Moderately soluble)
Consensus LogP	2.53	1.46
Synthetic Accessibility	3.14	3.56

**Fig. 12:**
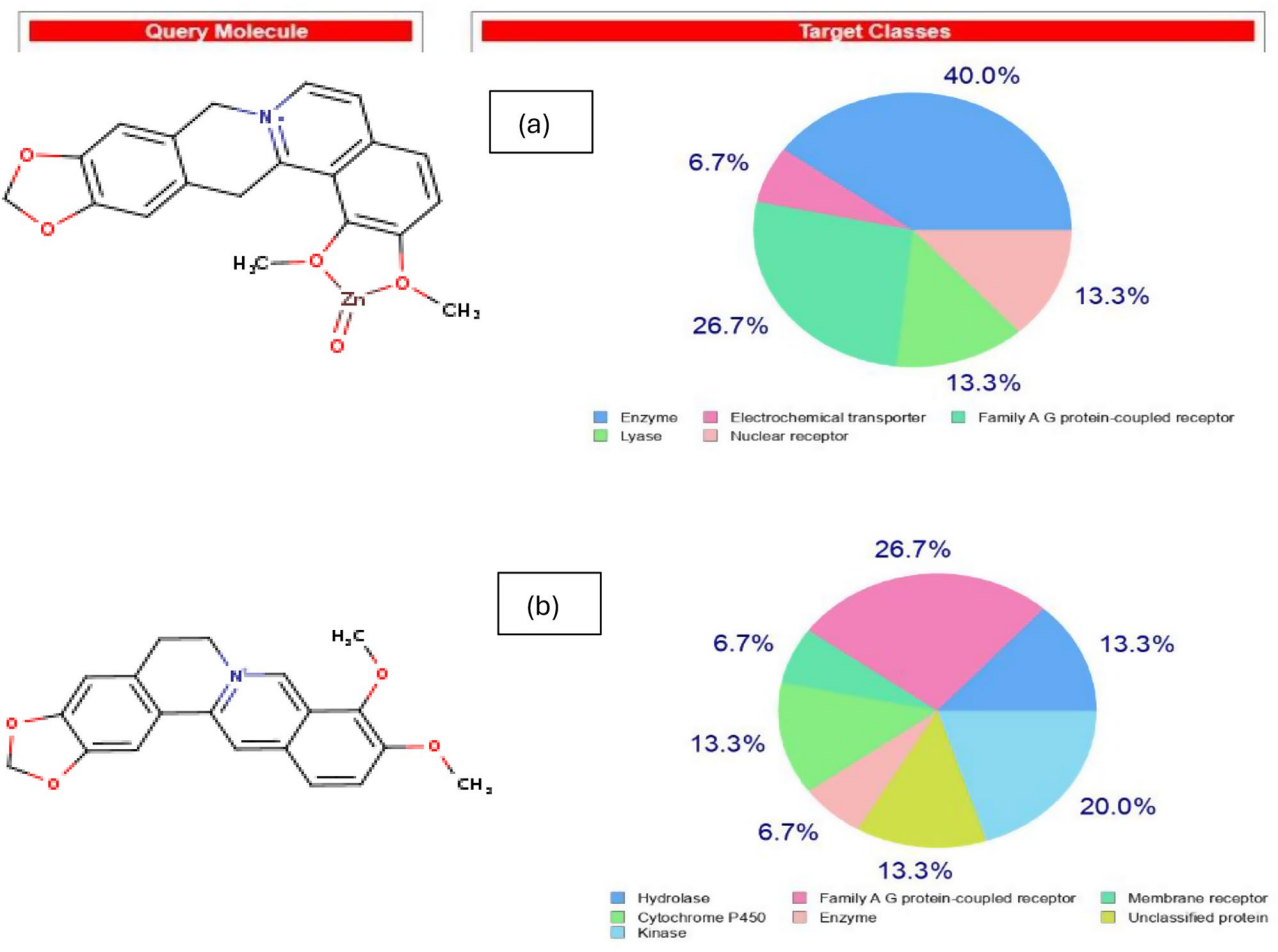
Target class distribution of both compounds (**a**) ZnO/Ber and (**b**) Berberine

Figure 12 shows that both compounds engage a broad spectrum of biological targets, including those relevant to inflammation, immune modulation, and airway remodeling. Moreover, Table 5 shows that both compounds target the same key inflammatory and immune pathway’ targets involved in COPD. While ZnO/Ber has superior effects toward carbonic anhydrases (CA9, CA12) which are important for pH regulation and mucus production.

**Table 5:** COPD-relevant targets for both Berberine and ZnO/Ber NPs

Target Type / Pathway	Berberine	ZnO/Ber	Notes
Muscarinic receptors (CHRM1, CHRM3)	√	√	Bronchoconstriction control
Adrenergic receptors (ADRA2B/C)	√	√	Airway tone regulation
Sigma opioid receptor (SIGMAR1)	√	√	Anti-inflammatory, neuroprotective
Cyclooxygenase (PTGS2)	√	√	Inflammation mediator
Carbonic anhydrases (CA9, CA12)	X	√	pH regulation, mucus production
Cytochrome P450s (CYP2D6, CYP11B1)	√	√	Drug metabolism, steroid synthesis
Nuclear receptors (NR3C1, PGR)	√	√	Steroid response, inflammation control
MAPK pathway kinases (MAPK14, MAPKAPK2)	√	√	Inflammatory signaling
NF-κB pathway (RELA)	√	√	Central to COPD inflammation
Transporters (SLC6A9, SLC1A3)	√	√	Neurotransmitter and amino acid transport

## Discussion

This study aimed to assess the therapeutic effect of ZnO/Ber NPs, which act as anti-inflammatory and potent antioxidant nanocomposites for treating ARDS. ARDS is characterized by lung inflammation [38], due to the overproduction of cytokines that associated with oxidative stress results in the accumulation of protein-rich edema in the alveoli, consequently resulting in hypoxemia that leads to ventilation‒perfusion mismatch [5]. Presently, existing treatments for ALI are largely ineffective and may result in severe side effects. Consequently, there is a pressing need to develop new therapeutic approaches that minimize these adverse effects [11].

In our study, LPS combined with nicotine was used to induce ARDS in a mouse model. LPS is well known for triggering the host inflammatory response through binding to TLR4. The activated TLR4 upon the binding of LPS stimulates the NF-κB signaling pathway, which leads to the production of proinflammatory mediators such as TNF-α and IL-1β [39] in addition to increasing IL-10 and IFN-γ [40] that leads to the recruitment of leukocytes and tissue damage.

In this study induction group showed an elevation in TREM-1 and it’s well known that LPS is a direct or indirect ligand for TREM-1, which is a transmembrane protein expressed on myeloid cells, such as monocytes and neutrophils,. and plays a crucial role in its activation [41]. TREM-1 activation leads to the amplification of the NF-κB inflammatory pathway and the overproduction of proinflammatory cytokines and chemokines [38]. In addition, TREM-1 increases the transepithelial infiltration of neutrophils into inflamed lungs [42] that consequently produces several cytotoxic products such as reactive oxygen species (ROS), granular enzymes such as myeloperoxidase (MPO), and proinflammatory cytokines that enhance the inflammatory process and finally contributes to lung tissue damage and dysfunction [22]. MPO has been shown to act as a local mediator of tissue damage and the subsequent inflammatory response. Numerous studies have reported that LPS-induced mice present a significant increase in the lung MPO level [12, 39], as evidenced by the present study. As a result of lung tissue damage, there is considerable loss of type II pneumocytes, which express ACE II. ACE II converts Ang II, which contributes to bronchoconstriction, cytokine expression, and cell apoptosis, promoting tissue injury [43], to Ang (1–7), which exerts anti-apoptotic and vasodilation effects [44]. ACE II has a protective effect on the lung. Hence, ACE II significantly decreased after LPS administration.

Nicotine accumulation in alveoli has a deleterious effect on the lungs [45]. Nicotine binding via α7nAChR enhances inflammatory responses by activating the NF-κB pathway. Furthermore, nicotine can lead to the accumulation of neutrophils and macrophages in the lung and disruption of the alveolar‒capillary barrier, leading to respiratory disorders [17].

In addition to inflammation and elevated cytokines, ARDS lung tissue showed increased MDA and disrupted oxidative balance, evidenced by reduced GSH and antioxidant enzymes (GPx, SOD, GST). Activated leukocytes generate excessive ROS, exceeding antioxidant defenses and causing oxidative stress [12, 18, 26, 39]. LPS and nicotine elaborate this by depleting GSH, impairing GPx activity, and promoting lipid peroxidation. SOD converts superoxide to H₂O₂, which GPx then reduces using GSH [46]. Elevated ROS also recruit neutrophils, reinforcing the cycle between inflammation and oxidative stress.

**Fig. 13:**
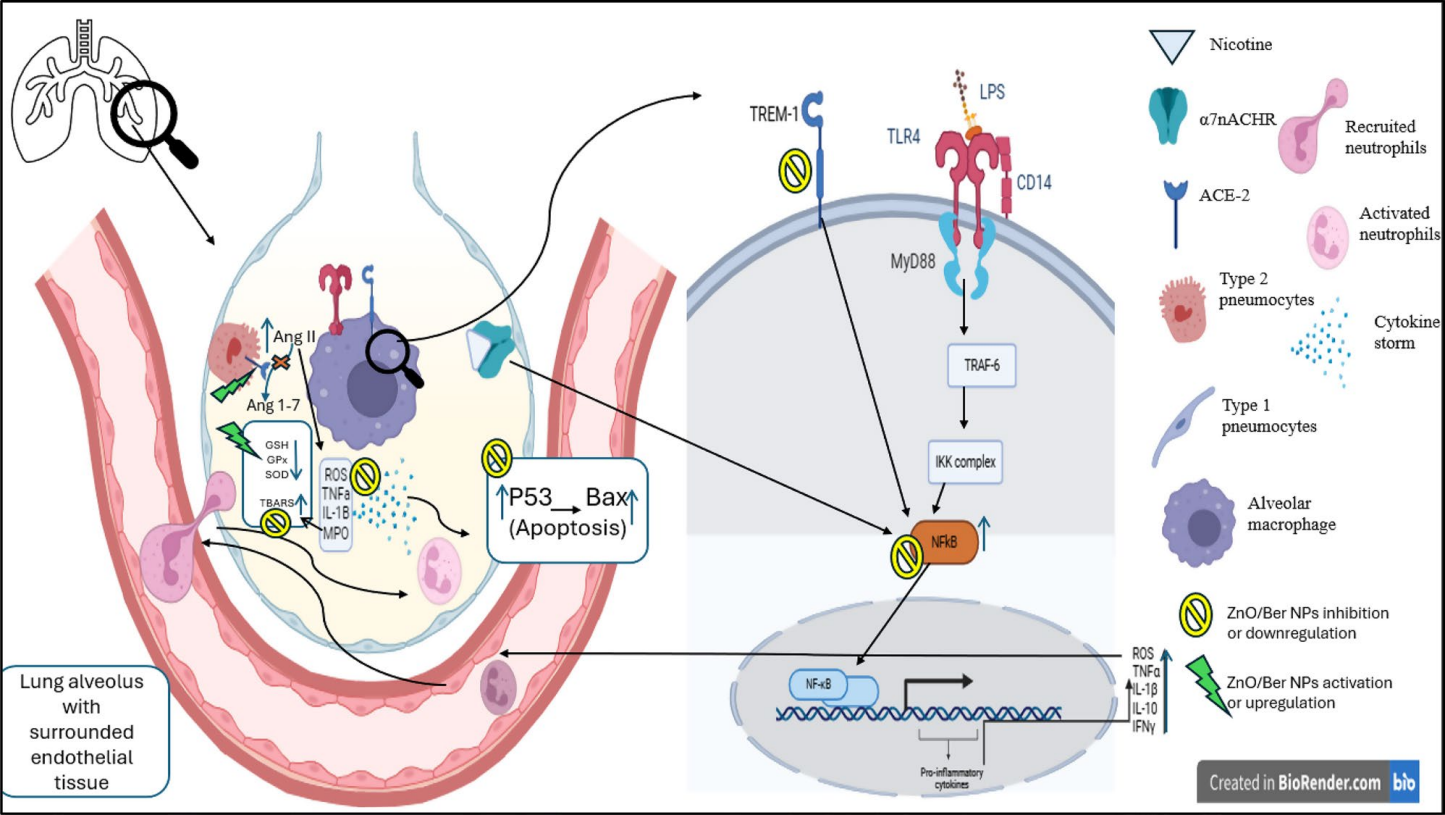
Schematic diagram of the mechanism of LPS + Nt-induced ARDS

Moreover, our results revealed that the p53 and Bax genes were overexpressed in LPS + Nt-induced mice. Studies suggest that elevated ROS and proinflammatory cytokines, especially TNF-α produced by alveolar leukocytes, trigger the activation of p53, which in turn upregulates the proapoptotic gene Bax and induces epithelial and endothelial cell apoptosis [47, 48]. In recent years, a growing body of research has shown that the onset of ARDS (ALI) is closely associated with inflammation, oxidative stress, and apoptosis [18, 49], as summarized in Fig. 13. In addition to the local lung inflammation shown by the histopathological results of lung tissues, as mentioned previously [37, 50], LPS + Nt induction resulted in systemic inflammation, as revealed by the significant increase in C-reactive protein in the sera of induced mice.

Current ARDS treatments are limited [51, 52], prompting interest in natural compounds with fewer side effects. Berberine (Ber) shows strong antioxidant and anti-inflammatory effects [53], including inhibition of TNF-α, IL-1β, MPO, and leukocyte infiltration in LPS- and cigarette smoke-induced pulmonary inflammation [54–56]The anti-inflammatory activity of Ber contributes to its ability to block the TLR4/NF-κB signaling pathway via the inhibition of inhibitor of nuclear factor kappa B (IkB) phosphorylation and degradation [57–59]. Moreover, Ber possesses an antioxidant effect that is associated with reduced MDA levels; increased levels of antioxidant enzymes (GPx, SOD and GST); and the ability to scavenge ROS [60, 61].

Nanoparticles (NPs), especially ZnO NPs, offer targeted delivery, biocompatibility, and anti-inflammatory potential [62, 63]. Additionally, it is used in inflammatory diseases, including ARDS, owing to the importance of zinc in sustaining and developing immune cells in the innate and adaptive immune systems. Disruption of zinc homeostasis leads to an increased risk of inflammation [64]. Our study showed ZnO NPs suppressed TREM-1 and MPO, increased ACE II, and had moderate antioxidant activity at 2.06 mg/kg. Fan et al. [65] reported dose-dependent antioxidant and anti-inflammatory effects of ZnO, reducing ROS and enhancing GSH and catalase.

Despite their excellent advantages, the use of Ber and ZnO NPs as individual therapeutic agents is limited due to their poor solubility, oral bioavailability, and cellular permeability, the moderate toxicity of ZnO NPs, and the high dose required for enhancing their antioxidant power. Hence, researchers have developed Ber-based nanoparticles [66] to overcome the limitations of Ber.

Our research group synthesized ZnO/Ber nanoparticles to enhance the therapeutic potential of Ber and ZnO and explored their anti-COVID-19 effects [1]. COVID-19 is associated with cytokine storms and ARDS, with ACE II as the key viral entry receptor [67]. Targeting ACE II–SARS-CoV-2 RBD interaction via conformational modulation offers a promising strategy. ZnO/Ber NPs showed superior antioxidant and anti-inflammatory effects over individual components, reducing TNF-α and IL-6 levels. They also bind stably to ACE II and the viral spike RBD, inhibiting viral entry. According to [68], ACE II has one active site and 3 allosteric sites (ASs), which are AS1, AS2, and AS3; from these sites, AS1 could be a druggable site.

Glucocorticoids are widely used in ARDS for their anti-inflammatory effects, mainly by suppressing cytokine expression [69]. While short-term high-dose methylprednisolone showed no mortality benefit [70], longer treatment durations (≥ 7 days) improved survival [71, 72]. However, corticosteroids may cause adverse effects like muscle weakness, GI bleeding, and metabolic disturbances [69, 73]. Berberine and ZnO NPs demonstrated anti-hyperglycemic effects [74, 75], and mice maintained stable weight (20–25 g). Future studies will compare long-term effects of ZnO/Ber NPs versus glucocorticoids.

In alignment with these findings, in silico molecular docking confirmed that ZnO/Ber NPs have strong antioxidant effects, with low binding energies to GPx (− 12.19 kcal/mol) and SOD (− 9.63 kcal/mol). ZnO/Ber binds GLU136(H) in GPx, a surface-exposed residue likely involved contribute to allosteric regulation or protein stability rather than direct catalysis [76]. In SOD, it interacts with GLN143, which guides superoxide into the active site and stabilizes copper [77, 78]. These interactions support the proposed enzyme-stabilizing and activity-modulating effects of ZnO/Ber NPs on both GPx and SOD.

In ACE II, GLN143 may influence protein conformation and potential interfacial interactions [79]. Therefore, the binding of ZnO/Ber NPs at this residue could influence the allosteric regulation or structural modulation of ACEII.

ZnO/Ber also binds ASP60 in TREM-1, potentially disrupting ligand recognition and inflammatory signaling because ASP60 is positioned within the Ig-like V-type domain and is involved in ligand and coreceptor interactions as well as electrostatic surface architecture [80]. Finally, interaction with GLU242 in MPO may inhibit its catalytic activity by interfering with substrate positioning and reactive intermediate stabilization [81].

In agreement with the in silico data, the well-characterized ZnO/Ber NPs had the most potent therapeutic potential effect in alleviating ARDS induced by LPS and nicotine compared with Ber, ZnO NPs alone, and their mixture. Thus, compared with the other tested compounds, the ZnO/Ber NPs had intense antioxidant activity, which was demonstrated by significantly decreased TBARS and increased GSH levels and GPx, GST, and SOD activities. Moreover, ZnO/Ber NPs exhibited powerful anti-inflammatory effects, as revealed by significant reductions in the concentrations of proinflammatory cytokines (IL-1β, TNF-α, IL-10, and IFNɣ), TREM-1and MPO, and NF-κB gene expression while increasing the ACE II concentration. Furthermore, ZnO/Ber NPs diminished the gene expression of p53 and Bax, thus inhibiting epithelial and endothelial cell apoptosis (Fig. 13).

The enhanced efficacy of the ZnO/Ber NPs suggests the successful mitigation of the bioavailability limitations of berberine. Poor aqueous solubility often hinders drug absorption in the gastrointestinal tract, reducing bioavailability and therapeutic effectiveness. Strategies to address this include solubility-enhancing additives or advanced formulations [82]. Nanotechnology offers a promising solution, with studies demonstrating a two-to-four-fold increase in the antimicrobial activity of berberine nanoparticles [83]. The improved bioavailability of ZnO/Ber NPs likely explains their superior antioxidant and anti-inflammatory effects compared with those of individual compounds or their physical mixture.

## Conclusion

The combination of LPS and nicotine effectively induced ARDS-like symptoms through activation of TLR4, TREM-1, and nicotinic receptors, leading to NF-κB–mediated inflammation, oxidative stress, cytokine overproduction, and alveolar damage. Treatment with ZnO/Ber NPs significantly mitigated these effects by downregulating TREM-1 and NF-κB, restoring ACEII expression, and reducing oxidative stress and inflammation. The ZnO/Ber nanocomplex showed superior protective efficacy compared to monotherapies or physical combinations, with potential to minimize glucocorticoid-related side effects. Further studies are recommended to explore this effect.

### Limitations of the work

- Higher doses should be studied to investigate possible side effects.
- Pharmacokinetics study should be performed to determine the maximum plasma concentration (Cmax) and maximum velocity (Vmax).
- Therapeutic effects on other mechanisms should be assessed.

## Supplementary Information

Below is the link to the electronic supplementary material.

Supplementary Material 1

## Data Availability

All the data generated or analyzed during this study are included in this published article.

## Abbreviations

ACE II: Angiotensin converting enzyme II
ALI: Acute lung injury
ARDS: Acute respiratory distress syndrome
Bax: Bcl-2-associated X protein
Ber: Berberine
GAPDH: Glyceraldehyde 3-phosphate dehydrogenase
HPLC: High Performance Liquid Chromatography
IFNγ: Interferon gamma
IL-10: Interleukin-10
IL-1β: Interleukin-1 beta
IL-6: Interleukin-6
LPS: Lipopolysaccharide
MPO: Myeloperoxidase
NF-κB: Nuclear factor kappa-B
Nt: Nicotine
ROS: Reactive oxygen species
TBARS: Thiobarbituric acid reactive substances
TLR4: Toll like receptor-4
TNF-α: Tumer necrosis factor-α
TREM-1: Triggering receptor expressed on myeloid cells − 1
ZnO NPs: Zinc oxide nanoparticles
ZnO/Ber NPs: Zinc oxide berberine nanoparticles
