# Supplementary material for: Therapeutic potential of zinc oxide/berberine nanoparticles in mitigating acute respiratory distress syndrome: in vivo and in silico approaches
Source: BMC Pharmacol Toxicol. 2025 Dec 1;26:205. doi: 10.1186/s40360-025-01036-5 (PMC12670808; doi:10.1186/s40360-025-01036-5)
Supplement: Supplementary file 1 — Supplementary Material 1 [file 40360_2025_1036_MOESM1_ESM.docx]

Table 1. Fifty conformations detected from the molecular docking simulation between berberine, ZnO/berberine and ZnO and 2R37 receptor.

| Parameter | Berberine with 2R37 receptor | | ZnO/berberine with 2R37 receptor | | ZnO with 2R37 receptor | |
| --- | --- | --- | --- | --- | --- | --- |
|  | Free energy of binding (Kcal/mol) | Inhibition constant Ki | Free energy of binding (Kcal/mol) | Inhibition constant Ki | Free energy of binding (Kcal/mol) | Inhibition constant Ki |
| 1 | -6.02 | 38.80 uM | -12.16 | 1.22 nM | -2.12 | 27.83 mM |
| 2 | -6.61 | 14.24 uM | -11.27 | 5.46 nM | -2.13 | 27.42 mM |
| 3 | -6.15 | 31.08 uM | -11.34 | 4.90 nM | -2.24 | 22.89 mM |
| 4 | -6.77 | 10.88 uM | -11.19 | 6.28 nM | -2.18 | 25.08 mM |
| 5 | -6.27 | 25.38 uM | -11.44 | 4.14 nM | -2.11 | 28.58 mM |
| 6 | -6.41 | 20.06 uM | -11.40 | 4.40 nM | -2.19 | 24.84 mM |
| 7 | -6.99 | 7.55 uM | -11.51 | 3.63 nM | -2.32 | 19.81 mM |
| 8 | -6.42 | 19.77 uM | -11.18 | 6.38 nM | -2.13 | 27.68 mM |
| 9 | -6.13 | 32.15 uM | -11.84 | 2.11 nM | -2.47 | 15.46 mM |
| 10 | -7.36 | 4.01 uM | -11.13 | 6.95 nM | -2.14 | 27.13 mM |
| 11 | -6.75 | 11.28 uM | -11.01 | 8.50 nM | -2.45 | 16.05 mM |
| 12 | -6.46 | 18.37 uM | -12.19 | 1.15 nM | -2.17 | 25.80 mM |
| 13 | -7.33 | 4.23 uM | -11.03 | 8.28 nM | -2.14 | 26.99 mM |
| 14 | -7.35 | 4.09 uM | -11.01 | 8.52 nM | -2.48 | 15.15 mM |
| 15 | -6.51 | 16.86 uM | -11.25 | 5.70 nM | -2.12 | 28.00 mM |
| 16 | -6.51 | 17.00 uM | -11.67 | 2.79 nM | -2.13 | 27.61 mM |
| 17 | -5.73 | 62.78 uM | -11.20 | 6.21 nM | -2.48 | 15.19 mM |
| 18 | -5.93 | 44.78 uM | -11.23 | 5.83 nM | -2.48 | 15.15 mM |
| 19 | -6.43 | 19.44 uM | -11.58 | 3.24 nM | -2.13 | 27.23 mM |
| 20 | -7.18 | 5.45 uM | -11.82 | 2.15 nM | -2.48 | 15.30 mM |
| 21 | -5.81 | 54.93 uM | -11.18 | 6.42 nM | -2.12 | 27.91 mM |
| 22 | -7.33 | 4.22 uM | -11.56 | 3.38 nM | -2.12 | 27.77 mM |
| 23 | -7.14 | 5.83 uM | -11.10 | 7.30 nM | -2.12 | 27.93 mM |
| 24 | -5.94 | 43.91 uM | -11.19 | 6.25 nM | -2.33 | 19.69 mM |
| 25 | -6.16 | 30.29 uM | -11.26 | 5.55 nM | -2.48 | 15.18 mM |
| 26 | -6.02 | 38.82 uM | -11.64 | 2.95 nM | -2.12 | 28.11 mM |
| 27 | -6.49 | 17.50 uM | -11.16 | 6.56 nM | -2.47 | 15.35 mM |
| 28 | -6.31 | 23.80 uM | -11.18 | 6.43 nM | -2.46 | 15.84 mM |
| 29 | -7.03 | 6.98 uM | -11.39 | 4.49 nM | -2.14 | 26.97 mM |
| 30 | -6.24 | 26.88 uM | -12.00 | 1.60 nM | -2.14 | 26.97 mM |
| 31 | -6.16 | 30.45 uM | -12.00 | 1.59 nM | -2.22 | 23.52 mM |
| 32 | -6.36 | 21.93 uM | -11.18 | 6.42 nM | -2.48 | 15.29 mM |
| 33 | -6.12 | 32.50 uM | -11.23 | 5.83 nM | -2.13 | 27.46 mM |
| 34 | -6.11 | 33.45 uM | -11.20 | 6.15 nM | -2.12 | 27.98 mM |
| 35 | -6.03 | 38.07 uM | -11.53 | 3.56 nM | -2.47 | 15.59 mM |
| 36 | -6.88 | 9.05 uM | -11.39 | 4.51 nM | -2.48 | 15.20 mM |
| 37 | -7.07 | 6.60 uM | -12.01 | 1.58 nM | -2.33 | 19.63 mM |
| 38 | -6.49 | 17.42 uM | -11.26 | 5.55 nM | -2.12 | 27.95 mM |
| 39 | -6.15 | 31.05 uM | -11.14 | 6.87 nM | -2.14 | 27.01 mM |
| 40 | -5.76 | 59.96 uM | -11.06 | 7.81 nM | -2.18 | 25.19 mM |
| 41 | -5.93 | 44.82 uM | -11.31 | 5.17 nM | -2.48 | 15.22 mM |
| 42 | -6.50 | 17.33 uM | -11.64 | 2.96 nM | -2.18 | 25.10 mM |
| 43 | -7.80 | 1.91 uM | -11.41 | 4.35 nM | -2.13 | 27.63 mM |
| 44 | -6.23 | 27.18 uM | -11.87 | 1.99 nM | -2.46 | 15.61 mM |
| 45 | -6.16 | 30.56 uM | -11.39 | 4.51 nM | -2.33 | 19.67 mM |
| 46 | -6.68 | 12.76 uM | -11.12 | 7.04 nM | -2.12 | 27.79 mM |
| 47 | -6.36 | 21.63 uM | -11.17 | 6.54 nM | -2.11 | 28.17 mM |
| 48 | -6.17 | 29.91 uM | -11.19 | 6.30 nM | -2.12 | 27.81 mM |
| 49 | -6.20 | 28.48 uM | -11.26 | 5.62 nM | -2.18 | 25.40 mM |
| 50 | -6.00 | 40.21 uM | -11.81 | 2.21 nM | -2.33 | 19.48 mM |

Table 2. Fifty conformations detected from the molecular docking simulation between berberine, ZnO/berberine and ZnO and 6M1D receptor.

| Parameter | Berberine with 6M1D receptor | | ZnO/berberine with 6M1D receptor | | ZnO with 6M1D receptor | |
| --- | --- | --- | --- | --- | --- | --- |
|  | Free energy of binding (Kcal/mol) | Inhibition constant Ki | Free energy of binding (Kcal/mol) | Inhibition constant Ki | Free energy of binding (Kcal/mol) | Inhibition constant Ki |
| 1 | -2.88 | 7.73 mM | -7.12 | 6.04 uM | -2.27 | 21.76 mM |
| 2 | -2.80 | 8.90 mM | -6.65 | 13.45 uM | -2.42 | 16.77 mM |
| 3 | -2.27 | 21.76 mM | -6.69 | 12.46 uM | -2.46 | 15.75 mM |
| 4 | -2.23 | 23.30 mM | -6.97 | 7.82 uM | -2.58 | 12.81 mM |
| 5 | -2.35 | 18.80 mM | -7.74 | 2.12 uM | -2.47 | 15.41 mM |
| 6 | -2.61 | 12.18 mM | -6.41 | 20.06 uM | -2.43 | 16.57 mM |
| 7 | -3.16 | 4.82 mM | -7.69 | 2.33 uM | -2.42 | 16.80 mM |
| 8 | -1.82 | 46.52 mM | -6.66 | 13.22 uM | -2.43 | 16.61 mM |
| 9 | -2.55 | 13.40 mM | -7.23 | 5.01 uM | -2.42 | 16.83 mM |
| 10 | -2.50 | 14.81 mM | -7.16 | 5.68 uM | -2.28 | 21.26 mM |
| 11 | -2.33 | 19.57 mM | -6.29 | 24.66 uM | -2.53 | 14.02 mM |
| 12 | -3.03 | 6.02 mM | -6.55 | 15.90 uM | -2.17 | 25.78 mM |
| 13 | -3.91 | 1.36 mM | -9.16 | 192.14 nM | -2.24 | 22.86 mM |
| 14 | -2.47 | 15.54 mM | -6.99 | 7.47 uM | -2.22 | 23.60 mM |
| 15 | -2.28 | 21.48 mM | -6.25 | 26.26 uM | -2.22 | 23.75 mM |
| 16 | -2.50 | 14.78 mM | -6.51 | 17.04 uM | -2.27 | 21.59 mM |
| 17 | -2.48 | 15.23 mM | -7.22 | 5.11 uM | -2.46 | 15.67 mM |
| 18 | -3.34 | 3.59 mM | -6.16 | 30.40 uM | -2.43 | 16.62 mM |
| 19 | -2.19 | 24.92 mM | -6.70 | 12.23 uM | -2.45 | 15.91 mM |
| 20 | -1.82 | 46.72 mM | -6.77 | 10.89 uM | -2.46 | 15.71 mM |
| 21 | -2.76 | 9.41 mM | -7.14 | 5.86 uM | -2.43 | 16.54 mM |
| 22 | -2.61 | 12.22 mM | -8.10 | 1.16 uM | -2.43 | 16.46 mM |
| 23 | -2.48 | 15.19 mM | -7.32 | 4.31 uM | -2.43 | 16.62 mM |
| 24 | -2.80 | 8.92 mM | -8.31 | 816.52 nM | -2.56 | 13.32 mM |
| 25 | -2.81 | 8.71 mM | -7.63 | 2.56 uM | -2.46 | 15.73 mM |
| 26 | -2.62 | 11.94 mM | -6.97 | 7.82 uM | -2.43 | 16.59 mM |
| 27 | -2.79 | 8.99 mM | -6.99 | 7.58 uM | -2.43 | 16.68 mM |
| 28 | -2.22 | 23.62 mM | -7.53 | 3.04 uM | -2.28 | 21.48 mM |
| 29 | -2.42 | 16.69 mM | -6.82 | 10.10 uM | -2.51 | 14.52 mM |
| 30 | -2.45 | 15.89 mM | -6.59 | 14.67 uM | -2.22 | 23.61 mM |
| 31 | -3.59 | 2.34 mM | -5.85 | 51.72 uM | -2.42 | 16.85 mM |
| 32 | -3.43 | 3.05 mM | -7.18 | 5.45 uM | -2.22 | 23.66 mM |
| 33 | -2.69 | 10.74 mM | -7.92 | 1.56 uM | -2.42 | 16.96 mM |
| 34 | -3.54 | 2.56 mM | -6.41 | 20.14 uM | -2.46 | 15.67 mM |
| 35 | -3.76 | 1.74 mM | -7.16 | 5.65 uM | -2.64 | 11.66 mM |
| 36 | -2.66 | 11.19 mM | -6.75 | 11.22 uM | -2.43 | 16.64 mM |
| 37 | -2.68 | 10.92 mM | -6.51 | 16.86 uM | -2.44 | 16.35 mM |
| 38 | -2.61 | 12.24 mM | -6.39 | 20.81 uM | -2.43 | 16.60 mM |
| 39 | -3.85 | 1.51 mM | -6.98 | 7.61 uM | -2.27 | 21.72 mM |
| 40 | -2.68 | 10.90 mM | -6.92 | 8.46 uM | -2.41 | 17.24 mM |
| 41 | -3.10 | 5.35 mM | -7.08 | 6.51 uM | -2.22 | 23.77 mM |
| 42 | -2.99 | 6.48 mM | -6.19 | 28.84 uM | -2.46 | 15.73 mM |
| 43 | -3.95 | 1.27 mM | -7.10 | 6.29 uM | -2.44 | 16.16 mM |
| 44 | -2.54 | 13.84 mM | -6.05 | 36.64 uM | -2.46 | 15.70 mM |
| 45 | -2.37 | 18.19 mM | -7.74 | 2.11 uM | -2.23 | 23.36 mM |
| 46 | -2.28 | 21.33 mM | -7.19 | 5.34 uM | -2.43 | 16.43 mM |
| 47 | -2.65 | 11.33 mM | -6.76 | 11.10 uM | -2.50 | 14.76 mM |
| 48 | -2.20 | 24.26 mM | -8.27 | 865.50 nM | -2.43 | 16.62 mM |
| 49 | -3.77 | 1.73 mM | -6.69 | 12.56 uM | -2.46 | 15.85 mM |
| 50 | -3.85 | 1.50 mM | -7.01 | 7.23 uM | -2.27 | 21.78 mM |

Table 3. Fifty conformations detected from the molecular docking simulation between berberine, ZnO/berberine and ZnO and 6WYZ receptor.

| Parameter | Berberine with 6WYZ receptor | | ZnO/berberine with 6WYZ receptor | | ZnO with 6WYZ receptor | |
| --- | --- | --- | --- | --- | --- | --- |
|  | Free energy of binding (Kcal/mol) | Inhibition constant Ki | Free energy of binding (Kcal/mol) | Inhibition constant Ki | Free energy of binding (Kcal/mol) | Inhibition constant Ki |
| 1 | -5.15 | 166.86 uM | -7.99 | 1.39 uM | -2.74 | 9.73 mM |
| 2 | -5.79 | 57.19 uM | -9.79 | 67.00 nM | -2.74 | 9.75 mM |
| 3 | -5.49 | 94.62 uM | -8.17 | 1.02 uM | -2.74 | 9.74 mM |
| 4 | -5.07 | 193.36 uM | -7.92 | 1.55 uM | -2.74 | 9.80 mM |
| 5 | -4.96 | 230.51 uM | -8.94 | 279.97 nM | -2.74 | 9.77 mM |
| 6 | -6.89 | 8.88 uM | -8.18 | 1.01 uM | -2.55 | 13.44 mM |
| 7 | -5.66 | 71.06 uM | -8.14 | 1.08 uM | -2.75 | 9.59 mM |
| 8 | -6.96 | 7.90 uM | -9.51 | 106.67 nM | -2.74 | 9.74 mM |
| 9 | -5.19 | 157.02 uM | -8.29 | 832.29 nM | -2.65 | 11.33 mM |
| 10 | -5.80 | 55.62 uM | -8.50 | 584.98 nM | -2.74 | 9.75 mM |
| 11 | -7.67 | 2.38 uM | -7.92 | 1.57 uM | -2.75 | 9.72 mM |
| 12 | -5.04 | 200.76 uM | -8.25 | 896.71 nM | -2.74 | 9.75 mM |
| 13 | -5.58 | 81.44 uM | -9.89 | 56.35 nM | -2.74 | 9.75 mM |
| 14 | -5.57 | 82.89 uM | -9.89 | 56.47 nM | -2.75 | 9.60 mM |
| 15 | -5.18 | 160.08 uM | -8.30 | 821.73 nM | -2.74 | 9.74 mM |
| 16 | -5.59 | 79.83 uM | -8.17 | 1.03 uM | -2.75 | 9.64 mM |
| 17 | -5.06 | 195.90 uM | -8.03 | 1.30 uM | -2.55 | 13.54 mM |
| 18 | -7.09 | 6.32 uM | -8.68 | 432.95 nM | -2.67 | 11.08 mM |
| 19 | -5.03 | 206.59 uM | -7.96 | 1.46 uM | -2.74 | 9.75 mM |
| 20 | -6.96 | 7.86 uM | -7.79 | 1.95 uM | -2.75 | 9.64 mM |
| 21 | -5.40 | 110.05 uM | -8.98 | 262.76 nM | -2.67 | 11.13 mM |
| 22 | -6.77 | 10.88 uM | -9.99 | 47.29 nM | -2.75 | 9.72 mM |
| 23 | -5.68 | 69.21 uM | -8.00 | 1.37 uM | -2.65 | 11.43 mM |
| 24 | -5.59 | 79.64 uM | -7.96 | 1.46 uM | -2.74 | 9.74 mM |
| 25 | -5.15 | 168.97 uM | -7.90 | 1.61 uM | -2.66 | 11.30 mM |
| 26 | -5.79 | 56.58 uM | -8.01 | 1.35 uM | -2.74 | 9.77 mM |
| 27 | -5.57 | 82.10 uM | -8.58 | 516.06 nM | -2.67 | 11.05 mM |
| 28 | -5.13 | 173.54 uM | -9.40 | 128.63 nM | -2.75 | 9.62 mM |
| 29 | -6.02 | 38.38 uM | -9.18 | 187.02 nM | -2.75 | 9.62 mM |
| 30 | -6.34 | 22.40 uM | -7.78 | 1.98 uM | -2.75 | 9.62 mM |
| 31 | -5.11 | 180.71 uM | -10.18 | 34.35 nM | -2.75 | 9.65 mM |
| 32 | -5.80 | 55.95 uM | -8.09 | 1.18 uM | -2.75 | 9.64 mM |
| 33 | -4.97 | 226.10 uM | -9.30 | 152.14 nM | -2.74 | 9.76 mM |
| 34 | -6.63 | 13.92 uM | -8.05 | 1.27 uM | -2.67 | 11.06 mM |
| 35 | -5.62 | 75.41 uM | -9.21 | 176.99 nM | -2.64 | 11.71 mM |
| 36 | -4.98 | 223.17 uM | -8.42 | 674.32 nM | -2.29 | 20.96 mM |
| 37 | -5.83 | 53.08 uM | -10.57 | 17.72 nM | -2.74 | 9.77 mM |
| 38 | -5.72 | 63.62 uM | -8.68 | 432.19 nM | -2.74 | 9.74 mM |
| 39 | -5.17 | 162.70 uM | -7.98 | 1.41 uM | -2.75 | 9.64 mM |
| 40 | -5.48 | 95.43 uM | -8.12 | 1.12 uM | -2.56 | 13.27 mM |
| 41 | -5.20 | 153.84 uM | -7.86 | 1.73 uM | -2.55 | 13.48 mM |
| 42 | -5.17 | 162.67 uM | -7.90 | 1.61 uM | -2.74 | 9.73 mM |
| 43 | -6.60 | 14.56 uM | -8.98 | 260.46 nM | -2.74 | 9.74 mM |
| 44 | -5.21 | 150.71 uM | -10.29 | 28.62 nM | -2.74 | 9.82 mM |
| 45 | -6.93 | 8.38 uM | -8.94 | 279.13 nM | -2.74 | 9.73 mM |
| 46 | -5.15 | 166.88 uM | -8.83 | 339.63 nM | -2.51 | 14.40 mM |
| 47 | -7.08 | 6.42 uM | -7.97 | 1.43 uM | -2.75 | 9.61 mM |
| 48 | -5.73 | 63.27 uM | -10.19 | 34.13 nM | -2.75 | 9.66 mM |
| 49 | -7.21 | 5.23 uM | -9.96 | 50.11 nM | -2.74 | 9.77 mM |
| 50 | -5.51 | 91.58 uM | -9.21 | 176.33 nM | -2.74 | 9.88 mM |

Table 4. Fifty conformations detected from the molecular docking simulation between berberine, ZnO/berberine and ZnO and 1PL4 receptor.

| Parameter | Berberine with IPL4 receptor | | ZnO/berberine with IPL4 receptor | | ZnO with IPL4 receptor | |
| --- | --- | --- | --- | --- | --- | --- |
|  | Free energy of binding (Kcal/mol) | Inhibition constant Ki | Free energy of binding (Kcal/mol) | Inhibition constant Ki | Free energy of binding (Kcal/mol) | Inhibition constant Ki |
| 1 | -3.98 | 1.21 mM | -6.39 | 20.70 uM | -2.77 | 9.36 mM |
| 2 | -3.79 | 1.65 mM | -8.21 | 957.91 nM | -2.24 | 22.62 mM |
| 3 | -4.97 | 227.70 uM | -7.94 | 1.51 uM | -2.88 | 7.75 mM |
| 4 | -3.95 | 1.28 mM | -6.33 | 22.81 uM | -2.87 | 7.81 mM |
| 5 | -3.79 | 1.68 mM | -9.63 | 86.90 nM | -2.52 | 14.16 mM |
| 6 | -5.06 | 193.84 uM | -6.42 | 19.83 uM | -2.84 | 8.28 mM |
| 7 | -3.51 | 2.69 mM | -6.56 | 15.62 uM | -2.87 | 7.93 mM |
| 8 | -3.77 | 1.71 mM | -6.56 | 15.55 uM | -2.52 | 14.16 mM |
| 9 | -4.04 | 1.10 mM | -5.93 | 44.97 uM | -2.53 | 14.05 mM |
| 10 | -3.78 | 1.71 mM | -7.93 | 1.54 uM | -2.73 | 9.92 mM |
| 11 | -4.17 | 879.94 uM | -7.91 | 1.59 uM | -2.77 | 9.39 mM |
| 12 | -4.20 | 836.68 uM | -6.97 | 7.74 uM | -2.70 | 10.45 mM |
| 13 | -6.20 | 28.37 uM | -6.82 | 10.09 uM | -2.87 | 7.93 mM |
| 14 | -5.55 | 85.62 uM | -6.88 | 8.98 uM | -2.51 | 14.51 mM |
| 15 | -5.18 | 158.55 uM | -7.79 | 1.94 uM | -2.53 | 14.07 mM |
| 16 | -3.88 | 1.43 mM | -6.91 | 8.61 uM | -2.73 | 9.98 mM |
| 17 | -4.35 | 650.96 uM | -6.19 | 29.21 uM | -2.52 | 14.22 mM |
| 18 | -3.81 | 1.61 mM | -9.13 | 204.21 nM | -2.52 | 14.15 mM |
| 19 | -4.33 | 671.44 uM | -6.72 | 11.88 uM | -2.52 | 14.21 mM |
| 20 | -4.68 | 370.49 uM | -7.16 | 5.65 uM | -2.69 | 10.74 mM |
| 21 | -4.07 | 1.04 mM | -6.93 | 8.36 uM | -2.84 | 8.25 mM |
| 22 | -5.13 | 173.04 uM | -8.22 | 948.80 nM | -2.52 | 14.21 mM |
| 23 | -4.31 | 697.93 uM | -6.92 | 8.50 uM | -2.71 | 10.37 mM |
| 24 | -4.42 | 575.65 uM | -6.91 | 8.68 uM | -2.74 | 9.87 mM |
| 25 | -3.82 | 1.57 mM | -6.68 | 12.72 uM | -2.53 | 14.06 mM |
| 26 | -3.92 | 1.35 mM | -6.51 | 16.85 uM | -2.88 | 7.75 mM |
| 27 | -4.06 | 1.06 mM | -6.73 | 11.61 uM | -2.45 | 15.97 mM |
| 28 | -5.51 | 91.43 uM | -6.57 | 15.37 uM | -2.81 | 8.78 mM |
| 29 | -3.49 | 2.75 mM | -7.63 | 2.54 uM | -2.52 | 14.23 mM |
| 30 | -6.00 | 39.90 uM | -6.56 | 15.52 uM | -2.79 | 8.98 mM |
| 31 | -3.91 | 1.35 mM | -7.66 | 2.42 uM | -2.52 | 14.17 mM |
| 32 | -4.09 | 999.90 uM | -6.35 | 22.22 uM | -2.77 | 9.39 mM |
| 33 | -4.01 | 1.15 mM | -6.36 | 21.87 uM | -2.88 | 7.80 mM |
| 34 | -4.21 | 822.54 uM | -6.80 | 10.41 uM | -2.52 | 14.16 mM |
| 35 | -6.25 | 26.25 uM | -6.71 | 11.98 uM | -2.53 | 14.06 mM |
| 36 | -6.74 | 11.40 uM | -7.17 | 5.53 uM | -2.69 | 10.72 mM |
| 37 | -3.70 | 1.95 mM | -6.70 | 12.20 uM | -2.84 | 8.29 mM |
| 38 | -3.80 | 1.64 mM | -6.40 | 20.36 uM | -2.22 | 23.79 mM |
| 39 | -4.90 | 258.02 uM | -6.23 | 26.93 uM | -2.53 | 14.07 mM |
| 40 | -4.10 | 981.32 uM | -6.89 | 8.91 uM | -2.85 | 8.17 mM |
| 41 | -4.21 | 814.09 uM | -7.14 | 5.88 uM | -2.23 | 23.18 mM |
| 42 | -4.12 | 950.58 uM | -9.41 | 125.97 nM | -2.81 | 8.69 mM |
| 43 | -4.12 | 953.94 uM | -6.86 | 9.44 uM | -2.81 | 8.75 mM |
| 44 | -4.51 | 494.82 uM | -6.60 | 14.47 uM | -2.53 | 14.04 mM |
| 45 | -5.22 | 148.66 uM | -6.69 | 12.48 uM | -2.71 | 10.26 mM |
| 46 | -3.80 | 1.63 mM | -7.05 | 6.85 uM | -2.51 | 14.45 mM |
| 47 | -3.60 | 2.28 mM | -6.18 | 29.72 uM | -2.52 | 14.17 mM |
| 48 | -3.77 | 1.71 mM | -7.52 | 3.10 uM | -2.51 | 14.36 mM |
| 49 | -4.04 | 1.10 mM | -7.75 | 2.07 uM | -2.52 | 14.29 mM |
| 50 | -3.98 | 1.21 mM | -6.68 | 12.76 uM | -2.79 | 8.97 mM |

Table 5.Ffifty conformations detected from the molecular docking simulation between berberine, ZnO/berberine and ZnO and 1Q8M receptor.

| Parameter | Berberine with 1Q8M receptor | | ZnO/berberine with 1Q8M receptor | | ZnO with 1Q8M receptor | |
| --- | --- | --- | --- | --- | --- | --- |
|  | Free energy of binding (Kcal/mol) | Inhibition constant Ki | Free energy of binding (Kcal/mol) | Inhibition constant Ki | Free energy of binding (Kcal/mol) | Inhibition constant Ki |
| 1 | -4.30 | 709.59 uM | +9.71 |  | -2.78 | 9.14 mM |
| 2 | -2.61 | 12.20 mM | -6.30 | 24.12 uM | -2.63 | 11.73 mM |
| 3 | -4.58 | 437.33 uM | -6.70 | 12.31 uM | -2.65 | 11.44 mM |
| 4 | -2.88 | 7.71 mM | -3.82 | 1.60 mM | -2.66 | 11.24 mM |
| 5 | -4.63 | 406.29 uM | -6.28 | 25.08 uM | -2.31 | 20.13 mM |
| 6 | -4.22 | 800.35 uM | +11.50 |  | -2.78 | 9.11 mM |
| 7 | -0.48 | 442.13 mM | -6.20 | 28.39 uM | -2.66 | 11.21 mM |
| 8 | -4.68 | 372.54 uM | -6.29 | 24.56 uM | -2.79 | 9.02 mM |
| 9 | -4.19 | 842.04 uM | -6.41 | 19.97 uM | -2.78 | 9.22 mM |
| 10 | -4.40 | 593.88 uM | +11.33 |  | -2.78 | 9.21 mM |
| 11 | -4.65 | 392.85 uM | -6.25 | 26.33 uM | -2.66 | 11.21 mM |
| 12 | -4.54 | 473.07 uM | -6.62 | 13.93 uM | -2.66 | 11.29 mM |
| 13 | -4.44 | 552.68 uM | -6.41 | 20.13 uM | -2.66 | 11.20 mM |
| 14 | -4.09 | 1.00 mM | +11.30 |  | -2.65 | 11.36 mM |
| 15 | -4.48 | 516.64 uM | +20.41 |  | -2.66 | 11.26 mM |
| 16 | -4.63 | 401.22 uM | -6.37 | 21.46 uM | -2.78 | 9.10 mM |
| 17 | -3.15 | 4.90 mM | -6.37 | 21.39 uM | -2.66 | 11.28 mM |
| 18 | -4.17 | 874.81 uM | -6.33 | 22.72 uM | -2.66 | 11.25 mM |
| 19 | -3.77 | 1.73 mM | -6.33 | 22.72 uM | -2.66 | 11.25 mM |
| 20 | -4.31 | 687.58 uM | +13.90 |  | -2.66 | 11.24 mM |
| 21 | -4.41 | 585.32 uM | +21.26 |  | -2.79 | 9.04 mM |
| 22 | -4.15 | 900.22 uM | -5.97 | 42.18 uM | -2.66 | 11.23 mM |
| 23 | -2.81 | 8.75 mM | -6.37 | 21.26 uM | -2.66 | 11.21 mM |
| 24 | -3.46 | 2.93 mM | +8.82 |  | -2.79 | 9.06 mM |
| 25 | -4.63 | 406.75 uM | -6.29 | 24.53 uM | -2.66 | 11.22 mM |
| 26 | -2.93 | 7.13 mM | -5.16 | 164.77 uM | -2.66 | 11.17 mM |
| 27 | -4.01 | 1.14 mM | +23.82 |  | -2.79 | 9.06 mM |
| 28 | -4.63 | 401.43 uM | -6.68 | 12.75 uM | -2.65 | 11.33 mM |
| 29 | -4.35 | 652.83 uM | -6.27 | 25.44 uM | -2.66 | 11.31 mM |
| 30 | -4.61 | 420.29 uM | -6.59 | 14.76 uM | -2.66 | 11.28 mM |
| 31 | -4.44 | 553.72 uM | -3.50 | 2.70 mM | -2.66 | 11.23 mM |
| 32 | -4.63 | 402.80 uM | -6.52 | 16.52 uM | -2.66 | 11.24 mM |
| 33 | -4.66 | 381.24 uM | -6.33 | 23.09 uM | -2.65 | 11.34 mM |
| 34 | -4.18 | 860.40 uM | -4.58 | 440.84 uM | -2.66 | 11.26 mM |
| 35 | -4.41 | 580.67 uM | -6.52 | 16.63 uM | -2.65 | 11.37 mM |
| 36 | -4.49 | 514.35 uM | -6.42 | 19.79 uM | -2.78 | 9.23 mM |
| 37 | -4.27 | 738.83 uM | -4.53 | 478.91 uM | -2.65 | 11.48 mM |
| 38 | -4.28 | 723.61 uM | -6.47 | 17.99 uM | -2.66 | 11.26 mM |
| 39 | -2.83 | 8.47 mM | -4.65 | 388.16 uM | -2.66 | 11.21 mM |
| 40 | -4.24 | 783.98 uM | -6.59 | 14.74 uM | -2.66 | 11.22 mM |
| 41 | -2.43 | 16.53 mM | +14.87 |  | -2.66 | 11.18 mM |
| 42 | -4.29 | 711.15 uM | -6.24 | 26.82 uM | -2.66 | 11.18 mM |
| 43 | +3.01 |  | -6.44 | 18.98 uM | -2.66 | 11.20 mM |
| 44 | -2.96 | 6.79 mM | -6.40 | 20.47 uM | -2.65 | 11.33 mM |
| 45 | -4.53 | 481.80 uM | -6.64 | 13.46 uM | -2.65 | 11.51 mM |
| 46 | +4.23 |  | -6.26 | 25.85 uM | -2.79 | 9.06 mM |
| 47 | -4.67 | 376.72 uM | -6.26 | 25.66 uM | -2.64 | 11.52 mM |
| 48 | -4.49 | 511.36 uM | -6.15 | 30.82 uM | -2.66 | 11.19 mM |
| 49 | -4.22 | 808.57 uM | -6.40 | 20.35 uM | -2.66 | 11.26 mM |
| 50 | -4.43 | 567.05 uM | -6.27 | 25.17 uM | -2.66 | 11.22 mM |
